# Dynamic response of microglia/macrophage polarization following demyelination in mice

**DOI:** 10.1186/s12974-019-1586-1

**Published:** 2019-10-17

**Authors:** Tianci Chu, Yi Ping Zhang, Zhisen Tian, Chuyuan Ye, Mingming Zhu, Lisa B. E. Shields, Maiying Kong, Gregory N. Barnes, Christopher B. Shields, Jun Cai

**Affiliations:** 1 Department of Pediatrics, Pediatric Research Institute, University of Louisville School of Medicine, Donald Baxter Building, Suite 321B, 570 S. Preston Street, Louisville, KY 40202 USA; 2 Norton Neuroscience Institute, Norton Healthcare, 210 East Gray Street, Suite 1102, Louisville, KY 40202 USA; 3 Department of Orthopedics, China-Japan Union Hospital of Jilin University, Changchun, 130033 People’s Republic of China; 4 Department of Pediatrics, The Second Affiliated Hospital and Yuying Children’s Hospital of Wenzhou Medical University, Wenzhou, 325027 People’s Republic of China; 5 Department of Radiology, University of Louisville School of Medicine, Louisville, KY 40202 USA; 6 Department of Bioinformatics and Biostatistics, University of Louisville School of Public Health and Information Sciences, Louisville, KY 40202 USA; 7 Department of Neurology, University of Louisville School of Medicine, Louisville, KY 40202 USA; 8 Department of Pharmacology and Toxicology, University of Louisville, Louisville, KY 40202 USA; 9 Department of Neurological Surgery, University of Louisville School of Medicine, Louisville, KY 40202 USA

**Keywords:** Multiple sclerosis, Focal demyelination model, Oligodendrocyte/oligodendrocyte progenitor cells, Microglia/macrophage, Neuroinflammation, Sensorimotor function, Diffusion tensor imaging

## Abstract

**Background:**

The glial response in multiple sclerosis (MS), especially for recruitment and differentiation of oligodendrocyte progenitor cells (OPCs), predicts the success of remyelination of MS plaques and return of function. As a central player in neuroinflammation, activation and polarization of microglia/macrophages (M/M) that modulate the inflammatory niche and cytokine components in demyelination lesions may impact the OPC response and progression of demyelination and remyelination. However, the dynamic behaviors of M/M and OPCs during demyelination and spontaneous remyelination are poorly understood, and the complex role of neuroinflammation in the demyelination-remyelination process is not well known. In this study, we utilized two focal demyelination models with different dynamic patterns of M/M to investigate the correlation between M/M polarization and the demyelination-remyelination process.

**Methods:**

The temporal and spatial features of M/M activation/polarization and OPC response in two focal demyelination models induced by lysolecithin (LPC) and lipopolysaccharide (LPS) were examined in mice. Detailed discrimination of morphology, sensorimotor function, diffusion tensor imaging (DTI), inflammation-relevant cytokines, and glial responses between these two models were analyzed at different phases.

**Results:**

The results show that LPC and LPS induced distinctive temporal and spatial lesion patterns. LPS produced diffuse demyelination lesions, with a delayed peak of demyelination and functional decline compared to LPC. Oligodendrocytes, astrocytes, and M/M were scattered throughout the LPS-induced demyelination lesions but were distributed in a layer-like pattern throughout the LPC-induced lesion. The specific M/M polarization was tightly correlated to the lesion pattern associated with balance beam function.

**Conclusions:**

This study elaborated on the spatial and temporal features of neuroinflammation mediators and glial response during the demyelination-remyelination processes in two focal demyelination models. Specific M/M polarization is highly correlated to the demyelination-remyelination process probably via modulations of the inflammatory niche, cytokine components, and OPC response. These findings not only provide a basis for understanding the complex and dynamic glial phenotypes and behaviors but also reveal potential targets to promote/inhibit certain M/M phenotypes at the appropriate time for efficient remyelination.

## Background

Remyelination is an endogenous regenerative process of damaged myelin sheaths in the adult central nervous system (CNS) that may restore saltatory conduction, metabolic transport, and trophic support [1–3]. Remyelination naturally occurs in multiple sclerosis (MS). However, it is often inadequate and results in progressive neurodegeneration that leads to chronic disabilities seen clinically [4–8]. This inadequate degree of remyelination is speculated to be related to the reduced recruitment of newly generated oligodendrocyte progenitor cells (OPCs) and the arrested differentiation of OPCs into mature oligodendrocytes (OLs) [1, 5, 9, 10]. As there is no efficient therapy to promote remyelination in MS, the underlying mechanisms of OPC/OL activity into the relevant cellular and molecular niches require further investigation to elucidate the process of demyelination-remyelination to create novel specific therapies.

Inflammatory responses within the CNS (neuroinflammation) is a major feature of MS [11]. The specific pathological, biochemical, and neurobehavioral consequences are related to the stimulus/injury type, microenvironment, and time-course [12]. As part of the immune system, microglia/macrophages (M/M) play a central role in neuroinflammation by antigen presentation, phagocytosis of apoptotic cells and cellular debris, as well as by the production of cytokines, chemokines, reactive oxygen species, secondary messengers, and growth/neurotrophic factors [12–14]. As a result of their role in the inflammatory response, M/M is implicated in the progression and alleviation of demyelination lesions [14–19]. This response is based not only on the presence of M/M but also on the phenotypic balance/switch of M/M that affects the severity of demyelination as well as the timing and efficiency of remyelination, presumably by alternating the behavior of OPCs [14–17, 20, 21]. The “classically activated” M1-phenotype is considered to be related to increased antigen presentation and production of pro-inflammatory cytokines and toxic molecules [16, 22], while the “alternatively activated” M2-phenotype is associated with remyelination by promoting OPC differentiation through the generated growth factors [16]. However, the temporal detail of M/M activation, polarization, and localization pattern of different M/M phenotypes, along with the response of OPC/OL at the same time-points, are poorly defined during the process of demyelination-remyelination.

The focal demyelination model is a useful tool to investigate the cellular response and molecular mechanisms during the demyelination-remyelination process as well as to screen and evaluate potential clinical treatments [23–25]. Focal demyelination models cannot recapitulate the complex pathophysiological changes and clinical effects of MS. However, localized injections of specific reagents create a highly reproducible lesion resembling the temporal profile of demyelination and spontaneous remyelination and creating predictable behavior dysfunction by generating a lesion in a predetermined location and precise size [24–27]. l-α-Lysophosphatidylcholine (lysolecithin or LPC) creates a focal demyelination lesion with a specific temporal profile and well-defined borders by directly dissolving phospholipid membranes and destroying myelin sheaths [27–29]. A significant loss of OLs and myelin occur within 1–3 days post-injection (dpi) after an LPC injection into the CNS white matter. Spontaneous remyelination is initiated between 7 and 14 dpi and is completed by 28 dpi depending on the lesion location and age of the animal [23, 29–31]. Unlike LPC that causes secondary inflammation after its cytotoxic effects, intraspinal injection of lipopolysaccharide (LPS), a toll-like receptor (TLR)-4 agonist, induces rapid neuroinflammation with the activation and polarization of M/M as well as secretion of pro-inflammatory cytokines, nitric oxide, and eicosanoids, resulting in prominent demyelination plaques with features of pattern III lesions of MS [26, 32, 33]. Demyelination appears at the injection site within 5–7 dpi and persists for 9–14 dpi with spontaneous remyelination occurring by 28 dpi [26, 32]. While LPC and LPS are commonly used to create focal demyelination models, little is known about their temporal characteristics, differences in M/M activation/polarization, and OPC/OL migration/differentiation during demyelination and spontaneous remyelination. Considering the crucial roles of M/M in neuroinflammation and the activity of OPC/OL in the demyelination-remyelination process, lack of this information impedes the understanding of underlying mechanisms leading to the specific characteristics of these lesions.

In this study, we examined the temporal patterns (histology and in vivo monitoring) and neurobehavioral changes during demyelination and spontaneous remyelination in two focal demyelination models (LPC and LPS) that represent different features of MS. The detailed dynamic characteristics of M/M activation/polarization, OPC/OL response, and relevant neuroinflammatory cytokines during the processes of demyelination-remyelination may provide novel targets for efficient remyelination in the treatment of MS.

## Methods

### Focal demyelination surgery

**Fig. 1:**
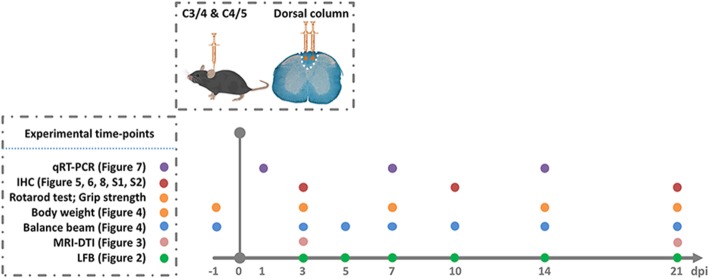
Graphic illustration of the experimental protocol at different time-points

Female C57BL/6 J mice (Jackson Laboratory, Bar Harbor, ME) were bred onsite and randomly assigned into three groups (control, LPC, and LPS groups) for surgery at 8 weeks of age (18.97 ± 1.02 g). Surgery to create a focal demyelination lesion was modified based on our previous reports [24, 25]. After being deeply anesthetized with 2,2,2-Tribromoethanol (300 mg/kg/i.p.; Sigma-Aldrich, St. Louis, MO), the C3-C5 spinal cord level was exposed by separating muscles and adipose tissues, removing the C3–5 laminae and ligamentum flavum, and opening dura using a surgical microscope. Focal demyelination lesions were created by stereotaxic injections into the dorsal funiculus at C3–4 and C4–5 using a pulled glass micropipette attached to a 10 μl Hamilton syringe (Hamilton Company, Reno, NV) connected to an infusion pump. Then, 1 μl of LPS (0.1 mg/ml, 0.01%; from *Salmonella enterica* serotype abortus equi; Sigma-Aldrich) or LPC (10 mg/ml, 1%; Type I; Sigma-Aldrich) was injected into both sides along the midline into the dorsal columns at a rate of 0.2 μl/min, and the micropipette was maintained in the dorsal columns for 2 min after each injection to avoid reflux. The control (CTR) group was injected with the same volume of sterile phosphate-buffered saline (PBS; Gibco, Carlsbad, CA) at the same rate as the vehicle group for both LPC and LPS groups. The muscle and adipose tissues were closed with a single suture, and the skin incision was closed with either rodent wound clips or suture (the latter used in mice scheduled for diffusion tensor imaging [DTI]). Each mouse was given saline (1 ml/s.c.; Nova-tech, Grand Island, NE) to prevent dehydration, buprenorphine hydrochloride (0.1 mg/kg/s.c.; Par Pharmaceuticals, Rochester, MI) for postoperative analgesia, and gentamycin (2 mg/kg/s.c.; Henry Schein Animal Health, Dublin, OH) for antibiotic prophylaxis every 12 h for 3 days. Performance of behavioral assessments and tissue collections followed a specific protocol (Fig. 1).

### Tissue processing

To obtain tissues for cryostat sectioning, mice were deeply anesthetized and killed by intracardiac perfusion with cold PBS (Gibco) and then 4% (w/v) paraformaldehyde (Sigma-Aldrich) at 3, 5, 7, 10, 14, and 21 dpi. The C2–T2 spinal cord segment was dissected and maintained on ice, followed by overnight post-fixation in 4% (w/v) paraformaldehyde at 4 °C. Spinal cords were cryoprotected in 20% (w/v) sucrose (RPI Research Products International Corp., Mount Prospect, IL) for 24 h at 4 °C and embedded in Tissue-Tek O.C.T. Compound (Sakura Finetek; Torrance, CA) for cryostat preparation. Transverse serial sections of 20 μm thickness were cut on a cryostat (Leica Biosystems Inc., Buffalo Grove, IL) for luxol fast blue (LFB) staining and immunohistochemistry. To collect tissues for ribonucleic acid (RNA) extraction, mice were deeply anesthetized and killed by intracardiac perfusion with cold PBS rinse at 1, 7, and 14 dpi. Fresh C3–C5 spinal cord segments (epicenter) were immediately dissected out on ice and snap-frozen in liquid nitrogen before being stored at − 80 °C. The frozen spinal cord segment was homogenized and lysed in RLT buffer supplemented with 2-mercaptoethanol (Sigma-Aldrich), followed by RNA extraction using the QIAshredder (Qiagen, Germantown, MD) and RNeasy Mini Kit (Qiagen).

### Luxol fast blue staining and quantitative analysis

**Fig. 2:**
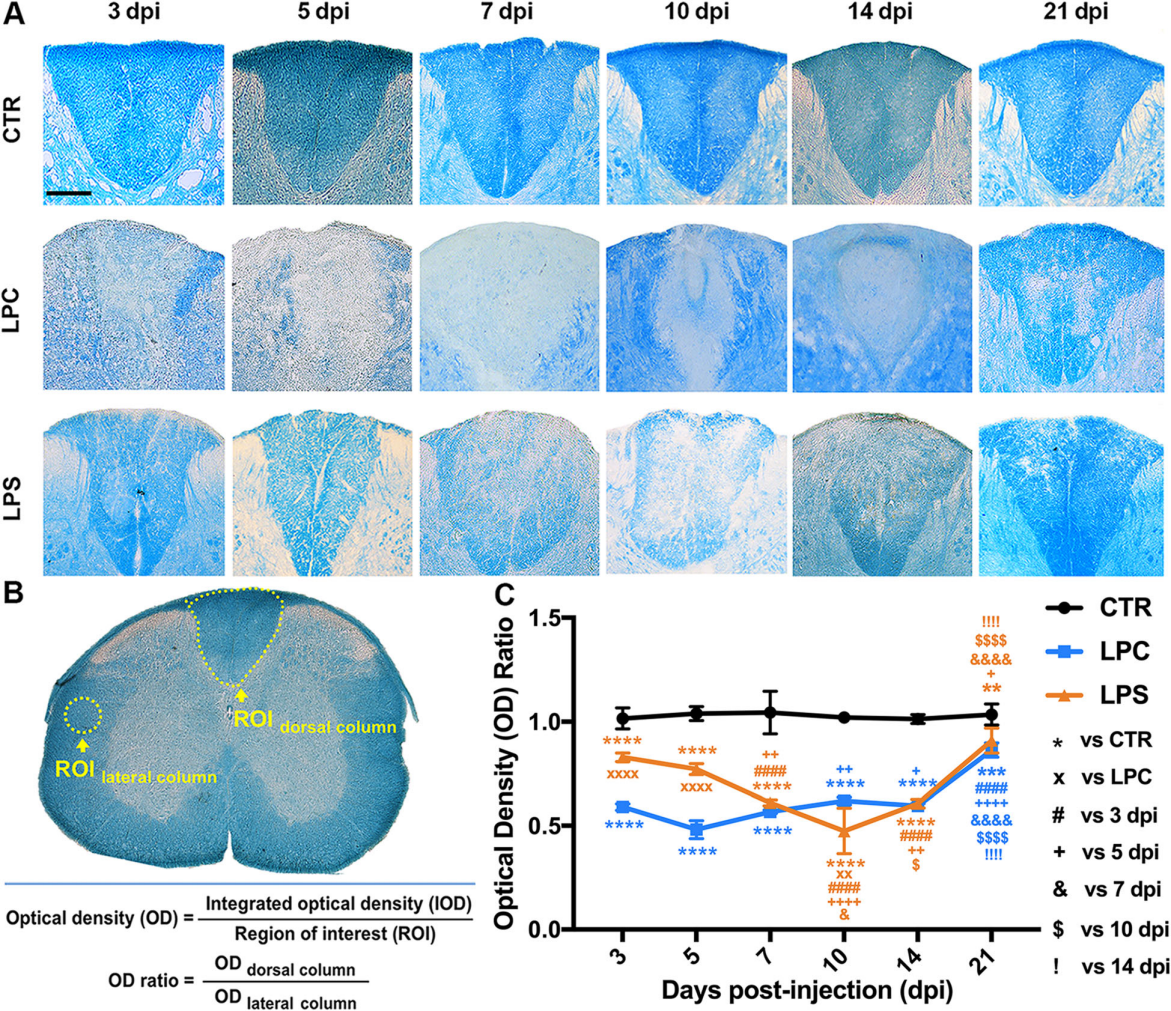
Temporal profile of the demyelinating lesions formed within the dorsal spinal cord after stereotaxic injections. **a** Representative light micrographs of LFB staining of the dorsal columns following PBS (CTR group), LPC, and LPS injections at different times. Scale bar = 200 μm. **b** Illustration of quantification method for LFB staining. OD ratio was calculated to measure the severity of demyelination with internal control. **c** Temporal changes in the OD ratio of LFB-stained dorsal lesions. For each time-point, the OD ratio was compared to the CTR group (**p < 0.01, ***p < 0.001, ****p < 0.0001) and the LPC group (××*p* < 0.01, ××××*p* < 0.0001); for each group, OD ratio was compared to 3 dpi (####*p* < 0.0001), 5 dpi (+*p* < 0.05, ++*p* < 0.01, ++++*p* < 0.0001), 7 dpi (&*p* < 0.05, &&&&*p* < 0.0001), 10 dpi ($*p* < 0.05, $$$$*p* < 0.0001), and 14 dpi (!!!!*p* < 0.0001). Data were collected from three animals per group at each time-point (*n* = 3 mice)

Myelin integrity was evaluated by LFB staining at 3, 5, 7, 10, 14, and 21 dpi as a standard histochemical method to stain myelin. Cryostat sections were incubated in 0.1% (w/v) LFB (Sigma-Aldrich) solution at 65 °C for 2 h after serial dehydration in ethanol solutions (Decon Labs, King of Prussia, PA). The sections were immersed in 0.05% (w/v) lithium carbonate (Sigma-Aldrich) solution and rinsed in 70% ethanol, followed by serial dehydration in ethanol solutions. The slides were cleansed in Xylene (Sigma-Aldrich) and mounted with Xylene-based mounting medium (Richard-Allan Scientific/Thermo Scientific, Waltham, MA) for microscopic visualization (Nikon Instruments, Inc., Melville, NY). To demonstrate the severity of the demyelination lesion, the integrated optical density (IOD) of the dorsal column at the epicenter level was measured and divided by the dorsal column area at the same level (i.e., ROI) to achieve its optical density (OD) value with ImageJ software (http://imagej.nih.gov/ij/; NIH, Bethesda, MD) (Fig. 2b, first equation). The OD ratio of the dorsal column OD value divided by the lateral column OD value on the same section was applied for normalization of the demyelination lesion in the dorsal column (Fig. 2b, second equation). The mean OD ratio of three epicenter sections was calculated for each mouse, which was considered as the observed value for group comparisons. Three mice per group at each time-point were used to examine group differences and time effects.

### Immunohistochemistry and quantitative analysis

Double immunohistochemistry on cryostat sections was performed based on our previous reports [34–36], and antibodies applied in this study are listed in Additional file 1: Table S1. Microwave-based antigen retrieval was performed in an antigen unmasking solution (citric acid based; Vector Laboratories, Burlingame, CA) for certain antibodies (as indicated in in Additional file 1: Table S1) to achieve better signals. For GFAP/CD68 staining, each staining area divided by the dorsal column area was quantified with ImageJ (positive-labeling area fraction), and mean positive-labeling area fraction of two epicenter fields (× 10 magnification) was calculated for each mouse. Mean values from three mice per group at each time-point were used to examine group differences and time effects. For the other co-staining, positive cells within the dorsal column area were manually counted using ImageJ and expressed as cell number per square millimeter (cell density). Mean cell density of three epicenter fields (× 20 magnification) was calculated for each mouse. Mean values from three mice per group at each time-point were used for statistical analysis on group differences and time effects.

### Real-time quantitative reverse transcription polymerase chain reaction

Gene expression was quantified by TaqMan gene expression assays (Applied Biosystems, Foster City, CA; as listed in in Additional file 2: Table S2) and TaqMan fast virus 1-step master mix (Applied Biosystems) following the manufacturer’s instructions. A total of 100 ng RNA from a fresh spinal cord segment (epicenter) was used per reaction. Real-time quantitative reverse transcription polymerase chain reaction (qRT-PCR) was conducted with Roche LightCycler 96 System (Roche, Branchburg, NJ), and Cq values were obtained and normalized to an endogenous reference (*β*-*actin*, *ACTB*) for relative quantification. Relative gene expression changes compared to the vehicle group at different time-points (1, 7, 14 dpi) were analyzed using the 2^−ΔΔCq^ method in a blinded manner [37, 38]. Data were collected and mean values were calculated from four mice per group at each time-point.

### Magnetic resonance imaging-diffusion tensor imaging

The MRI-DTI method and analysis were developed to detect the integrity of white matter (WM) microstructure based on the direction and magnitude of water diffusion [39–41]. Real-time monitoring was performed at the University of Louisville In Vivo Molecular Imaging Center using the Agilent 9.4 T/31 cm horizontal bore magnetic resonance imaging )(MRI system equipped with a 205/120 high-duty (HD) gradient coil (Agilent, Santa Clara, CA). As a noninvasive/live-monitoring technique that measures the direction and magnitude of water diffusion [42, 43], diffusion tensor imaging **(**DTI) targeting on mouse cervical spinal cords was performed at 3, 10, and 21 dpi after PBS, LPC, or LPS injection for detection and quantification of dorsal WM integrity. The affected segments (between C3–C5) were outlined as the epicenter for analysis. DTI metric parameters were obtained using DTIStudio (Johns Hopkins University, Baltimore, MD). Regions of interest (ROIs) were drawn and quantified using ImageJ software based on the anatomical landmarks of the MR images and mouse spinal cord atlas. DTI metrics of dorsal WM at the epicenter, including fractional anisotropy (FA), mean diffusivity (MD), axial diffusivity (λ_ll_; AD), and radial diffusivity (λ_⊥_; RD), were analyzed on the 78 × 78 μm^2^ in-plane pixel resolution images to characterize the changes in microstructural integrity, cellularity/edema/necrosis, axonal damage, and demyelination or dysmyelination. Mean FA, MD, AD, and RD of four epicenter images were calculated for each mouse. Mean values from three mice per group at each time-point were used for statistical analysis on group comparisons and time effects.

### Behavioral assessment

Demyelination lesions within the dorsal funiculi elicit possible functional alterations in mice. A series of behavioral assessments were conducted to evaluate mouse sensorimotor function at the pre-surgery stage (baseline) (− 1 dpi), early stage (3, 5 dpi), middle stage (7, 14 dpi), and late stage (21 dpi) to reveal the time-course of functional characteristics of LPC and LPS-induced dorsal demyelination. Body weight was recorded at the same time-points to monitor the body condition/health status of mice after surgery. The functional status was assessed by Rotarod performance, grip strength, and balance beam walking. Data were collected and mean values were calculated from nine mice per group with experimenter blinding.

#### Rotarod performance

This test evaluates locomotor function and coordination and was performed on the RotaRod (UgoBasile 7650 accelerating RotaRod, Varese, Italy) as described previously [34, 44]. In brief, a 2-day training/test regimen was followed with acceleration from 4 to 40 rpm in 300 s. The running time on the rod was recorded until the mouse fell off, and the average time of three trials on the test day was calculated as the Rotarod score. For each mouse, the Rotarod score to its baseline score (percent) was used for analysis to avoid functional variation between mice.

#### Forelimb/grip strength

This test was used to measure mouse forelimb/grip strength with a low-force testing system (ALMEMO, Woodland Hills, CA) and was conducted as described in our previous study [34]. The maximum pulling force (grams) of both forelimbs was digitally captured and recorded. The average value of three trials for each mouse was considered to be its forelimb/grip strength. The percentage of individual forelimb/grip strength to its baseline was calculated for analysis to avoid functional variation between mice.

#### Balance beam

This test evaluates fine sensorimotor function [45–47], and the method was modified based on previous reports [48, 49]. The mouse was allowed to cross an elevated (30 cm above the table top) square beam (50 cm in length) to reach the finish point, and the number of foot slips were recorded manually by two observers positioned on each side of the beam. Beam width ranged from 4 to 20 mm in five widths (4, 8, 12, 16, and 20 mm). Basic scores were based on whether the mouse could remain on the beam as follows: 4 mm = 25 points, 8 mm = 20 points, 12 mm = 15 points, 16 mm = 10 points, and 20 mm = 5 points. Pre-training was necessary for mice to achieve a stable and accurate performance. Four trials were performed on the test day with attention paid to (1) the beam width the mouse could transverse and (2) the number of foot slips in each trial (Final score = Basic score − Average number of foot slips for a total of four trials). The percentage of the individual balance beam score to its baseline was calculated for analysis to avoid functional variation between mice.

### Statistics

Data are expressed as mean ± standard deviation (SD). Comparisons between the groups (LPC vs. CTR; LPS vs. CTR; LPS vs. LPC) at different time-points were analyzed by two-way analysis of variance (ANOVA), followed by Tukey’s multiple comparisons test. Repeated measures two-way ANOVA was used for analyzing DTI metrics and behavioral assessments, followed by Tukey’s multiple comparisons test. The linear regression models and Pearson correlation coefficients were used to examine the correlations between two variables (behavior vs. myelin integrity; M/M subpopulation vs. myelin integrity). All statistical analyses were performed using GraphPad Prism 7.0 (GraphPad Software, San Diego, CA). The significance level was set at 0.05 for all comparisons.

## Results

### Temporal lesion patterns in LPC- and LPS-induced focal demyelination

As shown by LFB staining, the control group showed no signs of demyelination or changes in the OD ratio at any time-point. A rapid and robust loss of myelin occurred within the dorsal columns caused by LPC-induced demyelination which persisted until 21 dpi (Fig. 2a). Severe demyelination was observed throughout the early stage, and the OD ratio markedly declined at 3 dpi compared to the control group (0.59 in LPC vs. 1.02 in CTR, *p* < 0.0001), with an apparent trend toward a further decrease at 5 dpi (Fig. 2c; 0.48 in LPC vs. 1.04 in CTR, *p* < 0.0001). OD ratios remained decreased at the middle and late stages compared to the control group (Fig. 2c). However, gradual remyelination started at the middle stage (Fig. 2a, c; LPC: 0.62 at 10 dpi vs. 0.48 at 5 dpi, *p* < 0.01), and evident recovery was achieved at 21 dpi (Fig. 2a, c; LPC: 0.86 at 21 dpi vs. 0.48 at 5 dpi, *p* < 0.0001). For the LPS group, mild myelin loss was identified by LFB staining in the early stage with OD ratios statistically lower than those of the control group (3 dpi: 0.83 in LPS vs. 1.02 in CTR, *p* < 0.0001; 5 dpi: 0.77 in LPS vs. 1.04 in CTR, *p* < 0.0001) but higher than those of the LPC group (3 dpi: 0.83 in LPS vs. 0.59 in LPC, *p* < 0.0001; 5 dpi: 0.77 in LPS vs. 0.48 in LPC, *p <* 0.0001). During the middle stage, the severity of demyelination became more marked (Fig. 2a), and the OD ratios declined significantly compared to 3 dpi (LPS: 0.61 at 7dpi vs. 0.83 at 3 dpi, *p* < 0.0001), with the lowest value at 10 dpi (Fig. 2c; LPS: 0.47 at 10 dpi vs. 0.83 at 3 dpi, *p* < 0.0001). Similar to the LPC lesion, the OD ratio was statistically decreased compared to the control group (21 dpi: 0.91 in LPS vs. 1.04 in CTR, *p* < 0.01), significant remyelination was observed at 21 dpi (Fig. 2a, c; LPS: 0.91 at 21 dpi vs. 0.47 at 10 dpi, *p* < 0.0001).

### White matter integrity detected by MRI-DTI

**Fig. 3:**
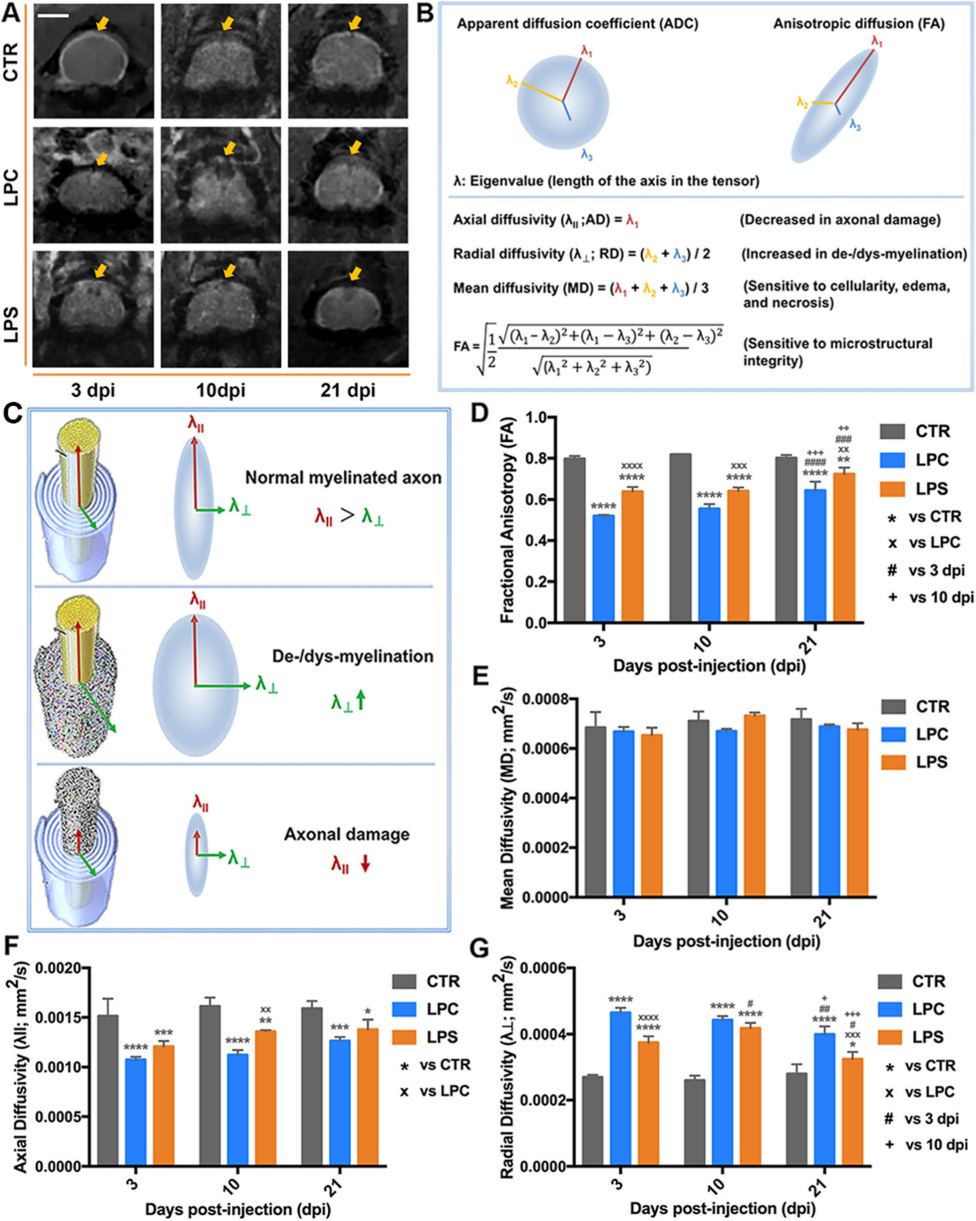
MRI-DTI images and metrics of the demyelination lesion. **a** Representative MRI images of the mouse spinal cord acquired at the lesion level at 3, 10, and 21 dpi. Arrow indicates the dorsal injection sites for CTR, LPC, and LPS groups. Scale bar = 1000 μm. **b** Illustration of DTI metrics. **c** Illustration of alternations in DTI metrics under pathological conditions. **d**–**g** DTI metrics FA, MD, λ_ll_, λ_⊥_, respectively of the dorsal spinal cord at the lesion level at 3, 10, and 21 dpi. For each time-point, the values were compared to the CTR group (*p < 0.05, **p < 0.01, ***p < 0.001, ****p < 0.0001) and the LPC group (××*p* < 0.01, ×××*p* < 0.001, ××××*p* < 0.0001); for each group, the values were compared to 3 dpi (#*p* < 0.05, ##*p* < 0.01, ###*p* < 0.001, ####*p* < 0.0001) and 10 dpi (+*p* < 0.05, ++*p* < 0.01, +++*p* < 0.001). Data were collected from three animals per group at each time-point (*n* = 3 mice)

In the representative trans-axial T2-weighted MR images (Fig. 3a), high signal intensity was seen in the LPC and LPS groups at 3 and 10 dpi, respectively. This might be a reflection of severe demyelination and/or inflammation-induced focal swelling in the spinal cord. FA was reduced at 3, 10, and 21 dpi after LPC injection (Fig. 3d; 3 dpi: 0.52 in LPC vs. 0.80 in CTR, *p* < 0.0001; 10 dpi: 0.55 in LPC vs. 0.82 in CTR, *p* < 0.0001; 21 dpi: 0.64 in LPC vs. 0.80 in CTR, *p* < 0.0001), indicating a persistent disruption of dorsal column integrity. FA in the LPC at 21 dpi had significantly improved compared to 3 and 10 dpi (0.64 at 21 dpi vs. 0.52 at 3 dpi, *p* < 0.0001; 0.64 at 21 dpi vs. 0.55 at 10 dpi, *p* < 0.001), corresponding to the remyelination/integrity recovery that was confirmed by LFB staining of histological sections (Fig. 2). Although the MD values of the LPC group was slightly decreased compared to the control group, there were no significant changes compared to the control group at any stage (Fig. 3e), suggesting that no significant edema or necrosis occurred in the dorsal columns. AD was reduced (Fig. 3f; LPC vs. CTR: 0.0011 vs. 0.0015 at 3 dpi, *p* < 0.0001; 0.0011 vs. 0.0016 at 10 dpi, *p* < 0.0001; 0.0013 vs. 0.0016 at 21 dpi, *p* < 0.001) while RD was elevated (Fig. 3g; LPC vs. CTR: 0.00046 vs. 0.00027 at 3 dpi, *p* < 0.0001; 0.00044 vs. 0.00026 at 10 dpi, *p* < 0.0001; 0.00040 vs. 0.00028 at 21 dpi, *p* < 0.0001) after LPC injection, indicating hindered water diffusion along the parallel axis of axons but increased water diffusion perpendicular to axons. This process was considered a consequence of demyelination or dysmyelination with axonal transport impairment [40, 50–52]. Similar to the change in FA, RD value at 21 dpi was markedly attenuated (0.00040 at 21 dpi vs. 0.00046 at 3 dpi, *p* < 0.01; 0.00040 at 21 dpi vs. 0.00044 at 10 dpi, *p* < 0.05); but AD was declined at all the stages with a recovery trend (Fig. 3f, g). These values reflect the severe demyelination or dysmyelination induced by LPC at the early stage, followed by a gradual improvement at the late stage; however, the axonal transport was impaired from the early stage but had not yet recovered at the late stage.

For the LPS group, FA was significantly reduced at 3 and 10 dpi (Fig. 3d; 3 dpi: 0.64 in LPS vs. 0.80 in CTR, *p* < 0.0001; 10 dpi: 0.64 in LPS vs. 0.82 in CTR, *p* < 0.0001). Although FA in LPS at 21 dpi was still lower than the control group (0.72 in LPS vs. 0.80 in CTR, *p* < 0.01), it had improved compared to 3 and 10 dpi (0.72 at 21 dpi vs. 0.64 at 3 dpi, *p* < 0.001; 0.72 at 21 dpi vs. 0.64 at 10 dpi, *p* < 0.01), consistent with the improved remyelination/integrity found in LFB staining results (Fig. 2). FA in LPS was higher than that in LPC at all stages (3 dpi: 0.64 in LPS vs. 0.52 in LPC, *p* < 0.0001; 10 dpi: 0.64 in LPS vs. 0.55 in LPC, *p* < 0.001; 21 dpi: 0.72 in LPS vs. 0.64 in LPC, *p* < 0.01), indicating that LPC causes more severe damage to the microstructural integrity. No statistical difference was detected in MD values between the control and LPS groups at any stage post-injection (Fig. 3e), suggesting that no significant edema or necrosis occurred in the dorsal columns. Compared to the control, RD in the LPS was elevated at 3 dpi and worsened at 10 dpi but significantly recovered at 21 dpi (LPS vs. CTR: 0.00037 vs. 0.00027 at 3 dpi, *p* < 0.0001; 0.00042 vs. 0.00026 at 10 dpi, *p* < 0.0001; 0.00032 vs. 0.00028 at 21 dpi, *p* < 0.05. LPS: 0.00032 at 21 dpi vs. 0.00037 at 3 dpi, *p* < 0.05; 0.00032 at 21 dpi vs. 0.00042 at 10 dpi, *p* < 0.001) while AD was reduced at all the stages only with a recovery trend (LPS vs. CTR: 0.0012 vs. 0.0015 at 3 dpi, *p* < 0.001; 0.0014 vs. 0.0016 at 10 dpi, *p* < 0.01; 0.0014 vs. 0.0016 at 21 dpi, *p* < 0.05) in the LPS group (Fig. 3f, g). These values reflect the LPS-induced demyelination or dysmyelination starting from 3 dpi, severe at 10 dpi, followed by recovery at 21 dpi; however, the axonal transport was impaired from 3 dpi but had not yet recovered at 21 dpi.

### Behavioral changes following the dorsal funicular demyelination

**Fig. 4:**
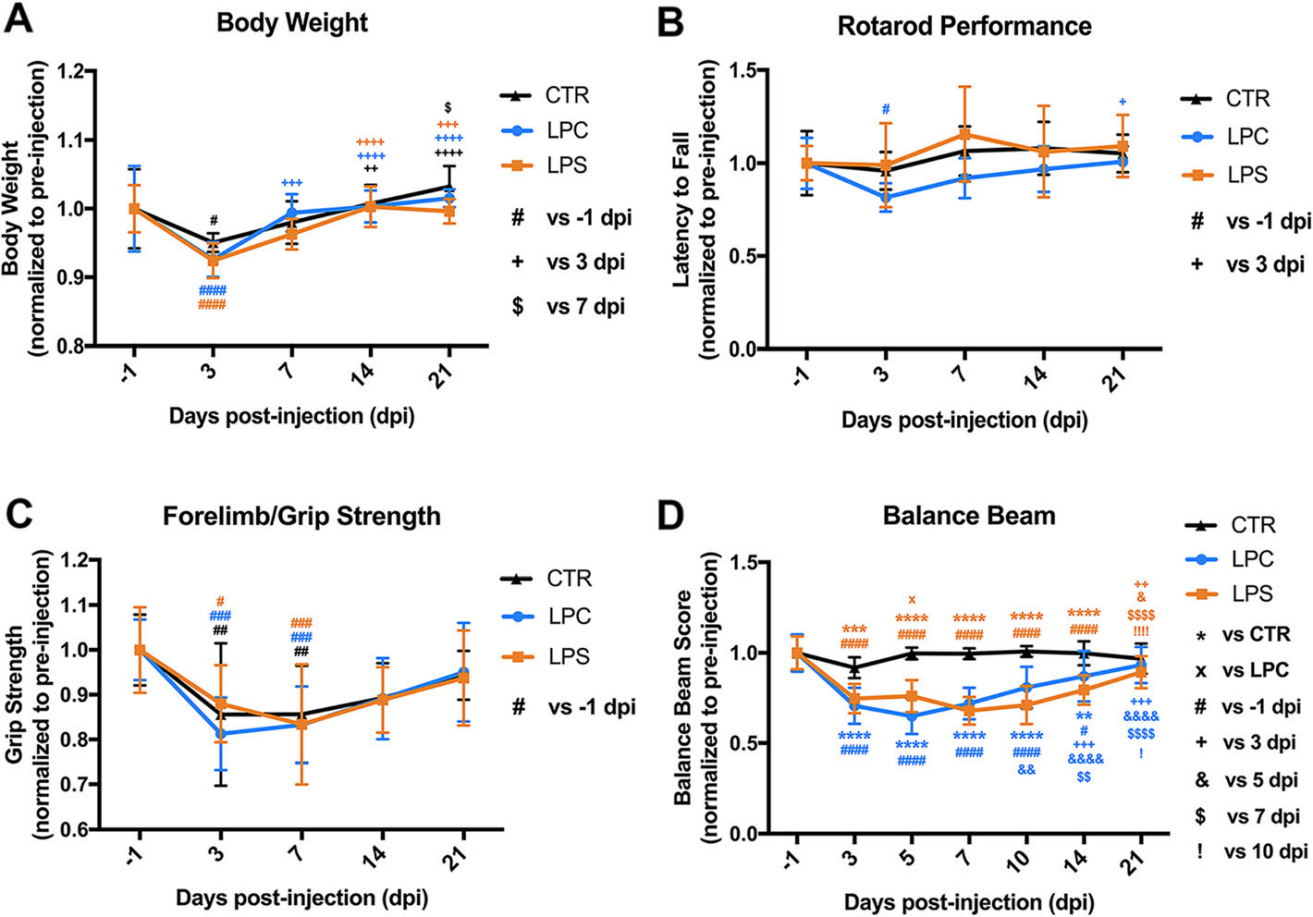
Temporal changes in the neurobehavioral performance of dorsal demyelination mouse models. **a** Body weight (normalized to each baseline at − 1 dpi) changes at different time-points after focal injection of PBS, LPC, and LPS into the dorsal column of the spinal cord. For each time-point, no statistical significance in body weight between the LPC/LPS groups and CTR group was noted; for each group, body weight was compared to − 1 dpi (#*p* < 0.05, ####*p* < 0.0001), 3 dpi (++*p* < 0.01, +++*p* < 0.001, ++++*p* < 0.0001), 7 dpi ($*p* < 0.05), and 14 dpi (no statistical significance for all groups). **b** Rotarod performance (normalized to each baseline at − 1 dpi) at different time-points. No statistical significance between the LPC or LPS group and CTR group were observed at any given time-point; for each group, no statistical significance between different time-points except when compared to − 1 dpi (#*p* < 0.05) and 3 dpi (+*p* < 0.05) in LPC group. **c** Grip strength (normalized to each baseline at − 1 dpi) at different time-points. For each time-point, no statistical significance in forelimb/grip strength was noted between the LPC/LPS groups and CTR group; for each group, no statistical significance between different time-points except when compared to − 1 dpi (#*p* < 0.05, ##*p* < 0.01, ###*p* < 0.001). **d** Balance beam score (normalized to each baseline at − 1 dpi) at different time-points. For each time-point, the balance beam score was compared to the CTR group (***p < 0.001, ****p < 0.0001) and the LPC group (×*p* < 0.05); for each group, balance beam score was compared to − 1 dpi (#*p* < 0.05, ####*p* < 0.0001), 3 dpi (++*p* < 0.01, ++++*p* < 0.0001), 5 dpi (&*p* < 0.05, &&*p* < 0.01, &&&&*p* < 0.0001), 7 dpi ($$*p* < 0.01, $$$$*p* < 0.0001), 10 dpi (!*p* < 0.05,!!!!*p* < 0.0001), and 14 dpi (no statistical significance for all groups). Data were collected from nine animals per group (*n* = 9 mice) with experimenter blinding

Mouse body weight showed an acute loss in all groups at 3 dpi compared to the pre-injection baseline (CTR: 0.95 at 3 dpi vs. 1.00 at pre-injection day, *p* < 0.05; LPC: 0.93 at 3 dpi vs. 1.00 at pre-injection day, *p* < 0.0001; LPS: 0.92 at 3 dpi vs. 1.00 at pre-injection day, *p* < 0.0001) but soon recovered. No significant difference was observed between groups at any time-point (Fig. 4a). Rotarod performance was assessed to evaluate mouse motor coordination and balance (Fig. 4b). Mice in the LPC group showed an apparent trend toward a decrease in the latency to fall from the Rotarod compared to the control mice at the early and middle stages. However, no statistical difference was found between the LPC or LPS group and CTR group, indicating that no major deficit in motor coordination function was induced by dorsal demyelination. Forelimb/grip strength was measured to show whether dorsal demyelination affected neuromuscular strength (Fig. 4c). In comparison to pre-injection baseline, grip strength showed an acute decrease in all groups (CTR: 0.86 at 3 dpi vs. 1.00 at pre-injection day, *p* < 0.01; LPC: 0.81 at 3 dpi vs. 1.00 at pre-injection day, *p* < 0.001; LPS: 0.88 at 3 dpi vs. 1.00 at pre-injection day, *p* < 0.05), all of which gradually resolved over time. Similar to the alterations in body weight, this initial decrease was probably caused by an acute reaction to surgery which could be further exacerbated by demyelination. No significant difference was detected between all groups. This data indicated that neuromuscular strength was not significantly affected by the dorsal demyelination lesions. To reveal fine deficits in mouse sensorimotor function, balance beam testing was conducted at several time-points (− 1, 3, 5, 7, 10, 14, and 21 dpi). The control group showed a consistent balance beam score without any statistical differences at all time-points, while both LPC and LPS groups exhibited significant decreases at all time-points except at 21 dpi (Fig. 4d). For the LPC group, the balance beam score dropped at 3 dpi (3 dpi: 0.71 in LPC vs. 0.92 in CTR, *p* < 0.0001; LPC: 0.71 at 3 dpi vs. 1.00 at pre-injection day, *p* < 0.0001), reached the lowest level at 5 dpi (5 dpi: 0.65 in LPC vs. 0.99 in CTR, *p* < 0.0001; LPC: 0.65 at 5 dpi vs. 1.00 at pre-injection day, *p* < 0.0001), and gradually improved up to 21 dpi when there was no significant difference compared to CTR or pre-injection baseline (21 dpi: 0.93 in LPC vs. 0.97 in CTR, *p* > 0.05; LPC: 0.93 at 21 dpi vs. 1.00 at pre-injection day, *p* > 0.05). Similarly, mice in the LPS group showed a significant decrease in balance beam score at 3 dpi (3 dpi: 0.75 in LPS vs. 0.92 in CTR, *p* < 0.001; LPS: 0.75 at 3 dpi vs. 1.00 at pre-injection day, *p* < 0.0001), reached its lowest level during the middle stage (7 dpi: 0.68 in LPS vs. 0.99 in CTR, *p* < 0.0001; LPS: 0.68 at 7 dpi vs. 1.00 at pre-surgery day, *p* < 0.0001; 10 dpi: 0.71 in LPS vs. 1.01 in CTR, *p* < 0.0001; LPS: 0.71 at 10 dpi vs. 1.00 at pre-injection day, *p* < 0.0001), and then gradually recovered by 21 dpi (21 dpi: 0.90 in LPS vs. 0.97 in CTR, *p* > 0.05; LPS: 0.90 at 21 dpi vs. 1.00 at pre-injection day, *p* > 0.05).

### Response of oligodendrocyte-linage cells following demyelination

**Fig. 5:**
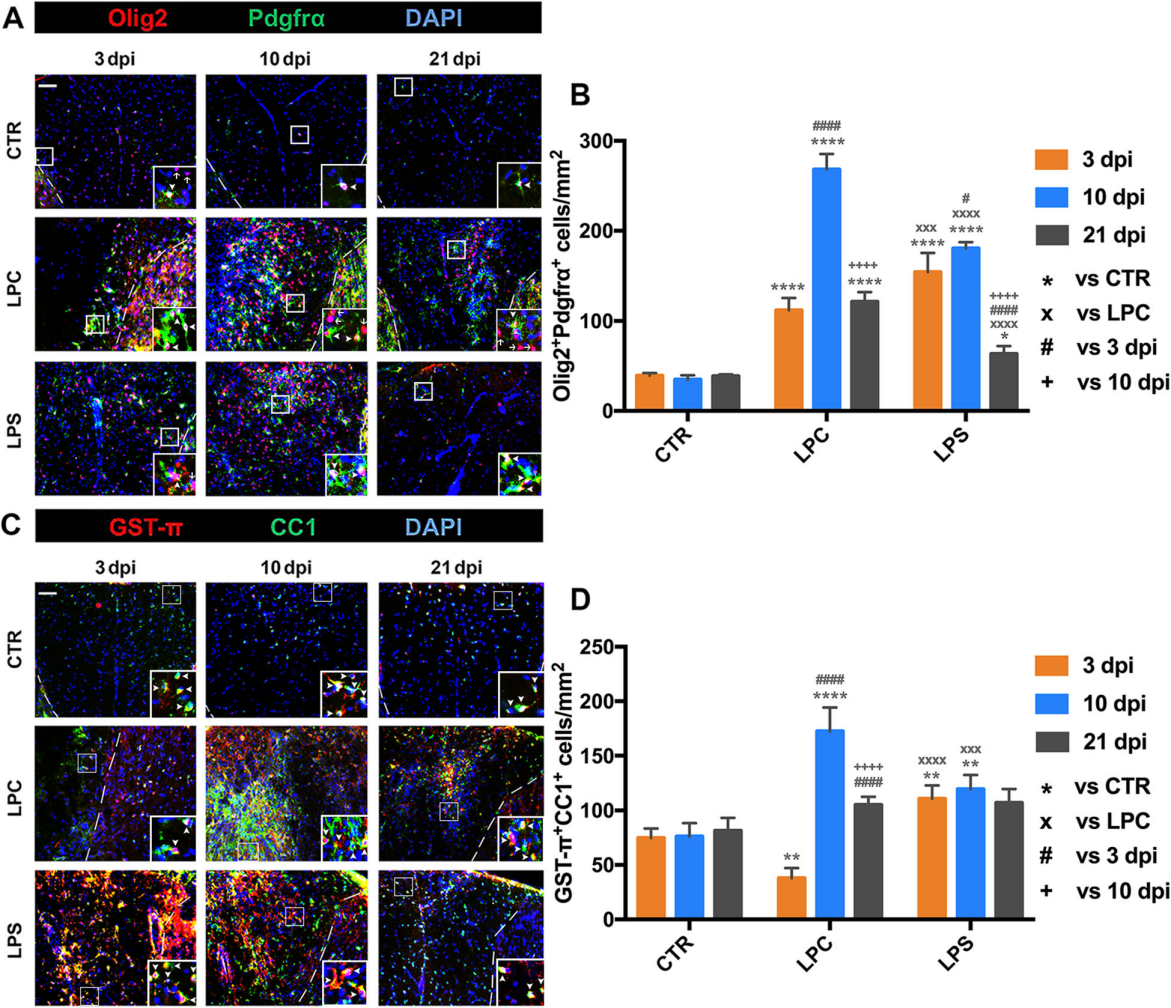
Dynamic response of oligodendrocyte-linage cells in localization and cell density following dorsal demyelination. **a** Representative photomicrographs of OPCs (Olig2^+^Pdgfrα^+^) in the dorsal demyelination area in CTR, LPC, and LPS groups at 3, 10, and 21 dpi. Dashed white line indicates the margins of the dorsal columns. Representative double labelling is indicated by arrowhead and single labelling is indicated by arrow in the inset for better view. Scale bar, 50 μm for panel figure; 20 μm for inset. **b** Quantification of OPC density within the dorsal column (Olig2^+^Pdgfrα^+^ cells/mm^2^) at the lesion site in the three groups at 3, 10, and 21 dpi. Cell density was compared to the CTR group (*p < 0.05, ****p < 0.0001) and the LPC group (×××*p* < 0.001, ××××*p* < 0.0001) at each given time-point; for each group, cell density was compared to 3 dpi (#*p* < 0.05, ####*p* < 0.0001) and 10 dpi (++++*p* < 0.0001). **c** Representative photomicrographs of mature OLs in the dorsal demyelination area (GST-π^+^CC1^+^) in CTR, LPC, and LPS groups at 3, 10, and 21 dpi (scale bar = 50 μm). Representative double labelling is indicated by arrowhead in the inset for better view (scale bar = 20 μm). **d** Quantification of mature OL density (GST-π^+^CC1^+^ cells/mm^2^) within the dorsal column at the lesion site in the three groups at 3, 10, and 21 dpi. The cell density was compared to the CTR group (**p < 0.01, ****p < 0.0001) and the LPC group (×××*p* < 0.001, ××××*p* < 0.0001) at each given time-point; for each group, cell density was compared to 3 dpi (####*p* < 0.0001) and 10 dpi (++++*p* < 0.0001). Data were collected from three animals per group at each time-point (*n* = 3 mice)

To examine the dynamic response of OPCs that determines the demyelination-remyelination process, cell densities of Olig2^+^Pdgfrα^+^ cells (OPCs; Fig. 5) and Pdgfrα^+^Ki67^+^ cells (proliferative OPCs; in Additional file 3: Figure S1 A and D) within the dorsal column were examined at early (3 dpi), middle (10 dpi), and late (21 dpi) stages. In LPC-induced focal lesions, adult Olig2^+^Pdgfrα^+^ OPCs aggregated along the margin around the epicenter at 3 dpi and further migrated into the lesion area at 10 dpi. In contrast, OPCs showed a diffuse pattern within the dorsal column and adjacent gray matter in LPS-induced lesions (Fig. 5a). OPCs presented in the control spinal cord at a low density throughout all time-points. After focal LPC or LPS injections, the density of OPCs significantly increased at 3 dpi (112.00 cells/mm^2^ in LPC vs. 39.18 cells/mm^2^ in CTR, *p* < 0.0001; 154.40 cells/mm^2^ in LPS vs. 39.18 cells/mm^2^ in CTR, *p* < 0.0001), reached the peak at 10 dpi (268.20 cells/mm^2^ in LPC vs. 34.90 cells/mm^2^ in CTR, *p* < 0.0001; 180.50 cells/mm^2^ in LPS vs. 34.90 cells/mm^2^ in CTR, *p* < 0.0001), and declined at 21 dpi (121.30 cells/mm^2^ in LPC vs. 38.72 cells/mm^2^ CTR, *p* < 0.0001; 63.27 cells/mm^2^ in LPS vs. 38.72 cells/mm^2^ in CTR, *p* < 0.05) (Fig. 5b). In addition to the OPC recruitment in focal demyelination lesions, proliferative OPCs were significantly increased within the dorsal column after 3 dpi (Additional file 3: Figure S1 A and D). In LPC-induced lesions, the proliferative Pdgfrα^+^Ki67^+^ OPCs erupted at 3dpi (Pdgfrα^+^Ki67^+^; 63.19 cells/mm^2^ in LPC vs. 0 cells/mm^2^ in CTR, *p* < 0.0001) and progressively decreased at 10 dpi (47.80 cells/mm^2^ in LPC vs. 0 cells/mm^2^ in CTR, *p* < 0.0001) and 21 dpi (21.56 cells/mm^2^ in LPC vs. 0 cells/mm^2^ in CTR, *p* < 0.001). The proliferative OPCs were also increased at 3 dpi (30.22 cells/mm^2^ in LPS vs. 0 cells/mm^2^ in CTR, *p* < 0.0001) in LPS-induced lesions. The peak of proliferation of OPCs occurred at 10 dpi (62.06 cells/mm^2^ in LPS vs. 0 cells/mm^2^ in CTR, *p* < 0.0001), followed by a decrease at 21 dpi (15.13 cells/mm^2^ in LPS vs. 0 cells/mm^2^ in CTR, *p* < 0.01).

To determine the temporal characteristics of OPC differentiation within the dorsal column, GST-π^+^CC1^+^ (differentiated/mature) oligodendrocytes within the dorsal column were counted at early (3 dpi), middle (10 dpi), and late stages (21 dpi) (Fig. 5c, d). GST-π^+^CC1^+^ cells were identified within the dorsal column at a relatively constant density with no significant changes between different time-points in the control group. For the LPC-induced lesion, the density of GST-π^+^CC1^+^ cells was significantly decreased at 3 dpi (37.75 cells/mm^2^ in LPC vs. 74.64 cells/mm^2^ in CTR, *p* < 0.01), with only few GST-π^+^CC1^+^ cells spared from damage along the dorsal column margin and some in the adjacent GM. At 10 dpi, the density was greatly increased within the demyelination area (172.30 cells/mm^2^ in LPC vs. 76.14 cells/mm^2^ in CTR, *p* < 0.0001), followed by a downregulation to the normal density at 21 dpi (105.00 cells/mm^2^ in LPC vs. 81.38 cells/mm^2^ in CTR, *p >* 0.05). Notably, the distribution of differentiated oligodendrocytes from the dorsal column margin into the demyelination center was similar to that of OPCs at 10 dpi (Fig. 5a). For the LPS-induced lesion, GST-π^+^CC1^+^ cells were increased at 3 dpi (110.80 cells/mm^2^ in LPS vs. 74.64 cells/mm^2^ in CTR, *p* < 0.01) that were scattered within the dorsal columns and maintained at the similar level at 10 dpi (119.50 cells/mm^2^ in LPS vs. 76.14 cells/mm^2^ in CTR, *p* < 0.01), but returned to the normal range at 21 dpi (106.90 cells/mm^2^ in LPS vs. 81.38 cells/mm^2^ in CTR, *p >* 0.05).

### Response of astrocytes and microglia/macrophages following demyelination

**Fig. 6:**
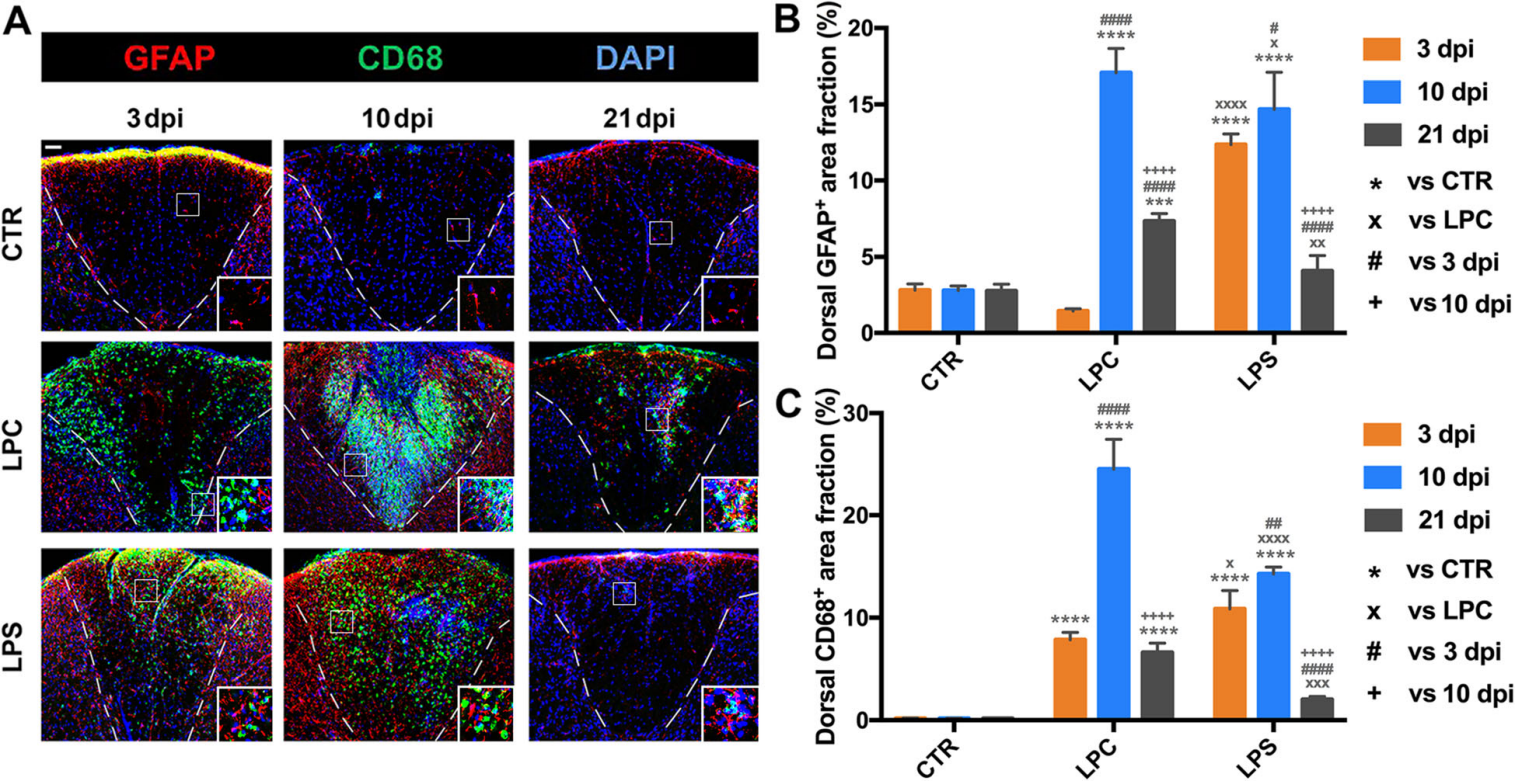
Dynamic glial activation after intraspinal injections of LPC and LPS. **a** Photomicrographs of immunostaining in the dorsal demyelination area showing the time course of astrocyte (GFAP^+^) and M/M (CD68^+^) activation at 3, 10, and 21 dpi. Dashed white line indicates the margins of the dorsal columns. Representative glial activation is better shown in the inset. Scale bar, 50 μm for panel figure; 25 μm for inset. **b** Quantification of astrocytic response within the dorsal columns (GFAP-labeling area fraction) at the lesion site in the three groups at 3, 10, and 21 dpi. GFAP-labeling area fraction was compared to the CTR group (***p < 0.001, ****p < 0.0001) and the LPC group (×*p* < 0.05, ××*p* < 0.01, ××××*p* < 0.0001) at each given time-point; for each group, GFAP^+^ area fraction was compared to 3 dpi (#*p* < 0.05, ####*p* < 0.0001) and 10 dpi (++++*p* < 0.0001). **c** Quantification of M/M response within the dorsal WM (CD68-labeling area fraction) at the lesion site in the three groups at 3, 10, and 21 dpi. The CD68-labeling area fraction was compared to the CTR group (****p < 0.0001) and the LPC group (×*p* < 0.05, ×××*p* < 0.001, ××××*p* < 0.0001) at each given time-point; for each group, CD68-labeling area fraction was compared to 3 dpi (##*p* < 0.01, ####*p* < 0.0001) and 10 dpi (++++*p* < 0.0001). Data were collected from three animals per group at each time-point (*n* = 3 mice)

To characterize the temporal activity of astrocytes and microglia/macrophages (M/M), fractions of GFAP- or CD68-labeling area within the dorsal columns were quantified at 3, 10, and 21 dpi after LPC or LPS injection (Fig. 6). The GFAP-labeling area fraction remained low in control mice at all time-points. GFAP-labeling area fraction was mildly decreased in the LPC mice but dramatically increased in the LPS mice at 3 dpi (1.45% in LPC vs. 2.80% in CTR, *p* > 0.05; 12.36% in LPS vs. 2.80% in CTR, *p* < 0.0001; 1.45% in LPC vs. 12.36% in LPS, *p* < 0.0001). At 10 dpi, GFAP-labeling area fraction reached the peak in the LPC mice (17.05% in LPC vs. 2.79% in CTR, *p* < 0.0001) and the LPS mice (14.68% in LPS vs. 2.79% in CTR, *p* < 0.0001). Both GFAP-labeling area fractions significantly decreased until 21 dpi, at which time it was still higher in LPC mice (7.32% in LPC vs. 2.76% in CTR, *p* < 0.001) but returned to the control range in LPS mice (4.07% in LPS vs. 2.76% in CTR, *p* > 0.05). The CD68 immunoreactivity was almost undetectable in control mice at all time-points. CD68-labeling area fraction was rapidly increased in both LPC and LPS mice at 3 dpi (7.84% in LPC vs. 0.17% in CTR, *p* < 0.0001; 10.85% in LPS vs. 0.17% in CTR, *p* < 0.0001; 7.84% in LPC vs. 10.85% in LPS, *p* < 0.05). At 10 dpi, CD68-labeling area fraction reached the peak in the LPC mice (24.51% in LPC vs. 0.17% in CTR, *p* < 0.0001), as well as in the LPS mice (14.29% in LPS vs. 0.17% in CTR, *p* < 0.0001). Similar to the changes in GFAP-labeling area fraction, both CD68-labeling area fractions were significantly reduced at 21 dpi, at which time it remained higher in LPC mice (6.58% in LPC vs. 0.16% in CTR, *p* < 0.0001) but returned to the control range in LPS mice (1.98% in LPS vs. 0.16% in CTR, *p* > 0.05).

In the LPC mice, cellular proliferation was robustly activated throughout all time-points in M/M (Additional file 3: Figure S1 B and E for Iba1^+^Ki67^+^; 3 dpi: 55.95 cells/mm^2^ in LPC vs. 0 cells/mm^2^ in CTR, *p* < 0.0001; 10 dpi: 145.30 cells/mm^2^ in LPC vs. 0 cells/mm^2^ in CTR, *p* < 0.0001; 21 dpi: 23.80 cells/mm^2^ in LPC vs. 0 cells/mm^2^ in CTR, *p* < 0.0001) and astrocytes (Additional file 3: Figure S1 C and F for GFAP^+^Ki67^+^; 3 dpi: 28.28 cells/mm^2^ in LPC vs. 0 cells/mm^2^ in CTR, *p* < 0.0001; 10 dpi: 26.87 cells/mm^2^ in LPC vs. 0 cells/mm^2^ in CTR, *p* < 0.0001; 21 dpi: 5.26 cells/mm^2^ in LPC vs. 0 cells/mm^2^ in CTR, *p* > 0.05). However, M/M showed much lower proliferation in LPS mice than in LPC mice (Additional file 3: Figure S1 B and E for Iba1^+^Ki67^+^; 3 dpi: 28.33 cells/mm^2^ in LPS vs. 0 cells/mm^2^ in CTR, *p* < 0.0001; 10 dpi: 24.50 cells/mm^2^ in LPS vs. 0 cells/mm^2^ in CTR, *p* < 0.0001; 21 dpi: 8.10 cells/mm^2^ in LPS vs. 0 cells/mm^2^ in CTR, *p* > 0.05. 3 dpi: 28.33 cells/mm^2^ in LPS vs. 55.95 cells/mm^2^ in LPC, *p* < 0.0001; 10 dpi: 24.50 cells/mm^2^ in LPS vs. 145.30 cells/mm^2^ in LPC, *p* < 0.0001; 21 dpi: 8.10 cells/mm^2^ in LPS vs. 23.80 cells/mm^2^ in LPC, *p* < 0.001). Astrocytes also significantly proliferated in LPS mice in the early and middle stages which was not observed at the late stage (Additional file 3: Figure S1 C and F for GFAP^+^Ki67^+^; 3 dpi: 35.51 cells/mm^2^ in LPS vs. 0 cells/mm^2^ in CTR, *p* < 0.0001; 10 dpi: 17.04 cells/mm^2^ in LPS vs. 0 cells/mm^2^ in CTR, *p* < 0.001; 21 dpi: 0 cells/mm^2^ in LPS vs. 0 cells/mm^2^ in CTR, *p* > 0.05).

Activated and proliferating M/M and astrocytes in LPC mice were observed mainly along the WM borders and directly adjacent to GM at 3 dpi. Then, GFAP^+^ astrocytes with enlarged processes were visible within the demyelination area around the lesion core while M/M were distributed throughout the entire demyelination lesion area. In contrast, all M/M and astrocytes in LPS mice were disbursed throughout the lesion area and adjacent GM throughout all time-points (Fig. 6a, Additional file 3: Figure S1 B and C).

### Time-course expression of pro-inflammatory and anti-inflammatory cytokines

**Fig. 7:**
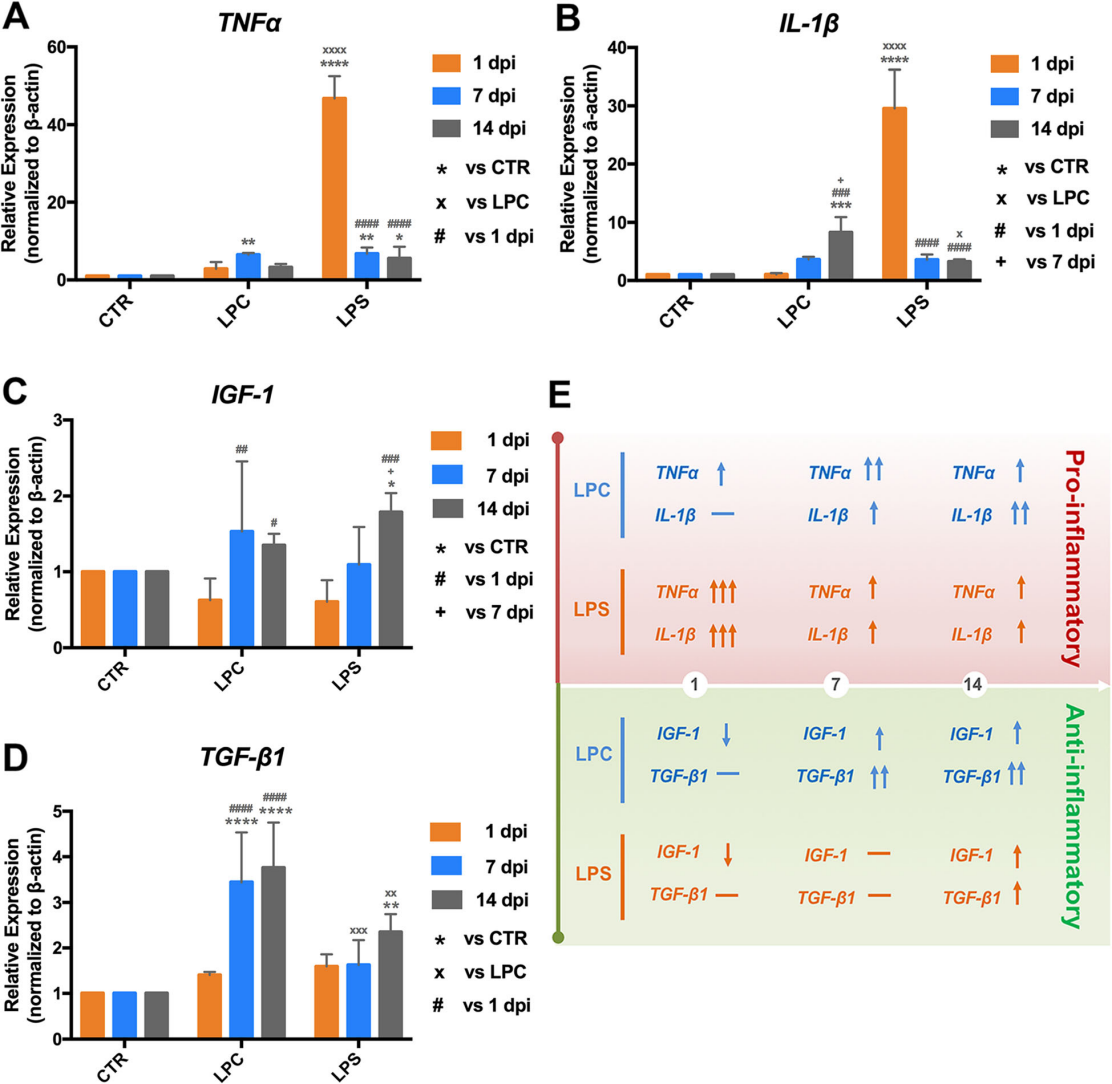
Time-course expression of pro-/anti-inflammatory cytokines at the injection level in LPC- and LPS-induced demyelination models. mRNA expressions of **a** *TNFα*, **b** *IL-1β*, **c** *IGF-1*, and **d** *TGF-β1* within the epicenter of the spinal cord lesion were examined by real-time qRT-PCR at 1, 7, and 14 dpi. For each time-point, relative gene expression was compared to the CTR group (*p < 0.05, **p < 0.01, ***p < 0.001, ****p < 0.0001) and the LPC group (×*p* < 0.05, ××*p* < 0.01, ×××*p* < 0.001, ××××*p* < 0.0001); for each group, relative gene expression was compared to 1 dpi (#*p* < 0.05, ##*p* < 0.01, ###*p* < 0.001, ####*p* < 0.0001) and 7 dpi (+*p* < 0.05). **e** Graphic summary of the temporal expression of pro-inflammatory and anti-inflammatory cytokines in LPC and LPS models. The direction of arrow indicates an up- or down trend of each cytokine, and the number of arrows indicate the relative magnitude of the expression change. Data were collected from four animals per group at each time-point (*n* = 4 mice)

To further confirm the inflammatory response and identify its potential role in the demyelination-remyelination process, the temporal expression levels of tumor necrosis factor alpha (*TNFα*) and *IL*-*1β* (pro-inflammatory cytokines) and *IGF*-*1* and *TGF*-*β1* (anti-inflammatory cytokines) within the epicenter were examined at 1, 7, and 14 dpi (Fig. 7). In the LPC mice, the relative expression ratio of *TNFα* was slightly increased at 1 dpi with no significant difference compared to the control group (2.89 in LPC vs. 1.00 in CTR, *p* > 0.05), then was significantly increased at 7 dpi (6.54 in LPC vs. 1.00 in CTR, *p* < 0.01), and returned to the control levels at 14 dpi (3.29 in LPC vs. 1.00 in CTR, *p* > 0.05) (Fig. 7a). The relative expression of *IL*-*1β* gradually increased with a significance difference found at 14 dpi (Fig. 7b; 8.29 in LPC vs. 1.00 in CTR, *p* < 0.001). Unlike LPC mice, the relative expression of both *TNFα* and *IL*-*1β* were dramatically upregulated and reached their peak levels at 1 dpi after LPS injection (Fig. 7a for *TNFα*: 46.79 in LPS vs. 1.00 in CTR, *p* < 0.0001; Fig. 7b for *IL*-*1β*: 29.54 in LPS vs. 1.00 in CTR, *p* < 0.0001). Both *TNFα* and *IL*-*1β* expression levels were then greatly downregulated at later stages, but *TNFα* expression remained moderately higher while *IL*-*1β* expression was not statistically different compared to the control (Fig. 7a for *TNFα*; 7 dpi: 6.76 in LPS vs. 1.00 in CTR, *p* < 0.01; 14 dpi: 5.59 in LPS vs. 1.00 in CTR, *p* < 0.05. Fig. 7b for *IL-1β*; 7 dpi: 3.57 in LPS vs. 1.00 in CTR, *p* > 0.05; 14 dpi: 3.24 vs. 1.00 in CTR, *p* > 0.05). However, the temporal changes in the expression of *IGF*-*1* and *TGF*-*β1* were similar in LPC and LPS mice (Fig. 7c, d): slightly decreased or equivalent compared to the control mice at 1 dpi and gradually increased at later stages.

### Temporal characteristics of M/M polarization and localization

**Fig. 8:**
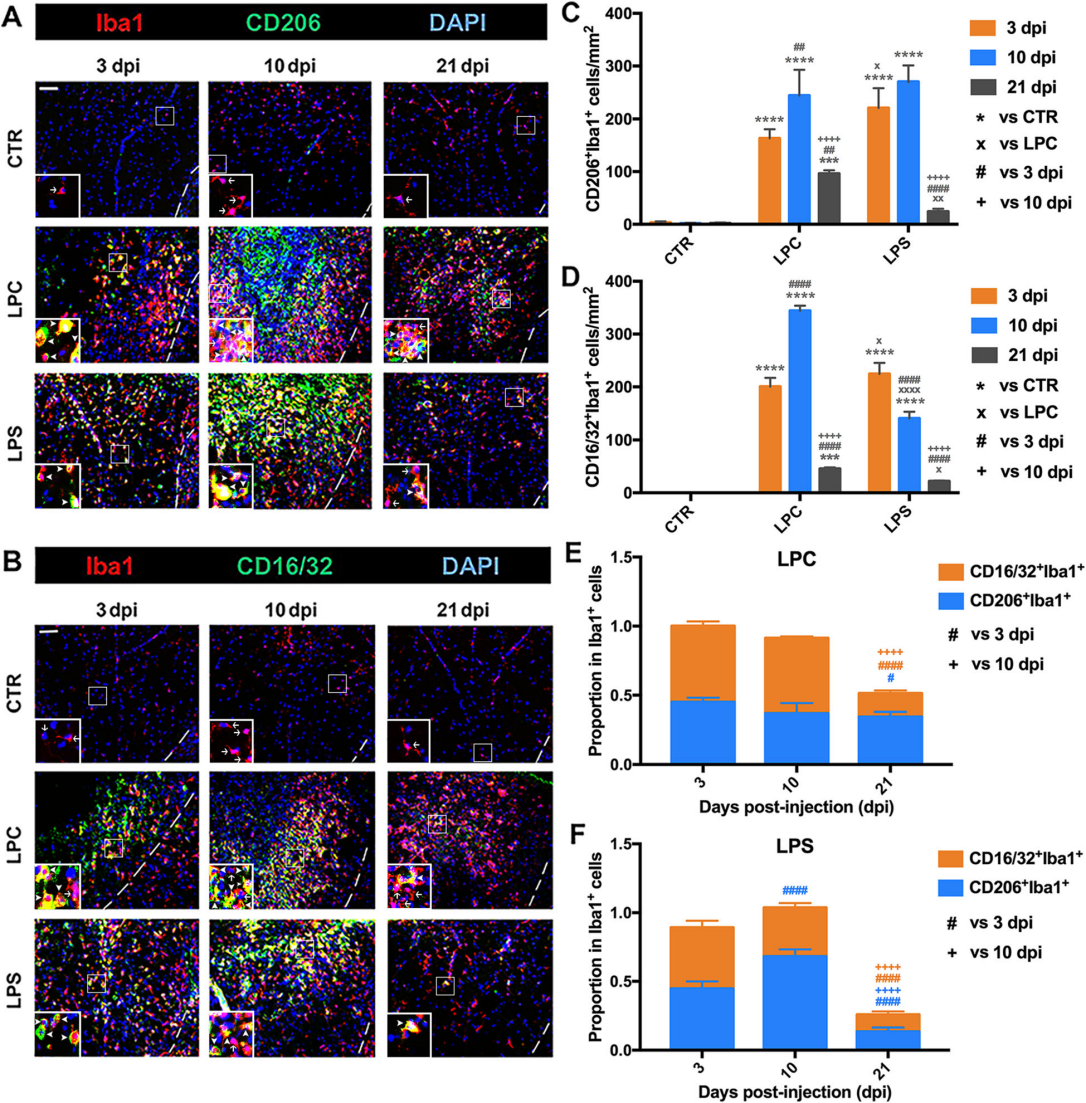
Temporal characteristics of M/M polarization and localization in LPC- and LPS-induced dorsal demyelination models. **a**, **b** Representative photomicrographs of **a** M2 (CD206^+^Iba1^+^) and **b** M1 (CD16/32^+^Iba1^+^) in the dorsal demyelination area in CTR, LPC, and LPS groups at 3, 10, and 21 dpi. Dashed white line indicates the margins of the dorsal columns. Representative double labelling is indicated by arrowhead and single labelling is indicated by arrow in the inset for better view. Scale bar, 50 μm for panel figure; 20 μm for inset. **c**, **d** Quantification of cell densities of **c** M2 (CD206^+^Iba1^+^ cells/mm^2^) and **d** M1 (CD16/32^+^Iba1^+^ cells/mm^2^) within the dorsal columns at the lesion site in the three groups at 3, 10, and 21 dpi. Cell density was compared to the CTR group (***p < 0.001, ****p < 0.0001) and the LPC group (×*p* < 0.05, ××*p* < 0.01, ××××*p* < 0.0001) at each given time-point; for each group, cell density was compared to 3 dpi (##*p* < 0.01, ####*p* < 0.0001) and 10 dpi (++++*p* < 0.0001). **e**, **f** Proportions of M2 (CD206^+^Iba1^+^) and M1 (CD16/32^+^Iba1^+^) in M/M (Iba1^+^) within the dorsal columns at the lesion site in **e** LPC and **f** LPS models at 3, 10, and 21 dpi. The cell proportion was compared to 3 dpi (#*p* < 0.05, ####*p* < 0.0001) and 10 dpi (++++*p* < 0.0001). Data were collected from three animals per group at each time-point (*n* = 3 mice)

To determine the timing of M/M polarization and localization pattern during the process of demyelination and remyelination, the densities of CD206^+^Iba1^+^ (M2) and CD16/32^+^Iba1^+^ (M1) cells at 3, 10, and 21 dpi were quantified within the dorsal column (Fig. 8). These findings were further confirmed by staining of Arg1^+^Iba1^+^ (M2) and CD86^+^Iba1^+^ (M1) cells (Additional file 4: Figure S2). M2 (CD206^+^Iba1^+^ or Arg1^+^Iba1^+^) or M1 (CD16/32^+^Iba1^+^ or CD86^+^Iba1^+^) cells were rarely detected throughout all time-points in the control mice. In the LPC mice, robust increases in the densities of CD206^+^Iba1^+^ cells (163.00 cells/mm^2^ in LPC vs. 3.32 cells/mm^2^ in CTR, *p* < 0.0001) while CD16/32^+^Iba1^+^ cells (200.40 cells/mm^2^ in LPC vs. 0 cells/mm^2^ in CTR, *p* < 0.0001) were observed within the spared dorsal margin at 3 dpi. Although both densities increased at 10 dpi (CD206^+^Iba1^+^: 244.70 cells/mm^2^ in LPC vs. 1.39 cells/mm^2^ in CTR, *p* < 0.0001; CD16/32^+^Iba1^+^: 344.20 cells/mm^2^ in LPC vs. 0 cells/mm^2^ in CTR, *p* < 0.0001), different localization patterns were noted within the demyelination lesion at this time-point of initial remyelination. CD206^+^Iba1^+^ cells were concentrated within the demyelination lesion core (Fig. 8a), surrounded by oligodendrocyte-linage cells (Fig. 5a, c), astrocytes (Fig. 6a), and other M/M cells, including CD16/32^+^Iba1^+^ cells (Fig. 8b). At 21 dpi, both CD206^+^Iba1^+^ cells and CD16/32^+^Iba1^+^ cells were evenly distributed within the remaining lesion area, and their densities were both significantly reduced (especially the CD16/32^+^Iba1^+^ cells) but still higher than those in the control mice (CD206^+^Iba1^+^: 96.10 cells/mm^2^ in LPC vs. 1.95 cells/mm^2^ in CTR, *p* < 0.001; CD16/32^+^Iba1^+^: 45.43 cells/mm^2^ in LPC vs. 0 cells/mm^2^ in CTR, *p* < 0.001). In LPS mice, significant increases in the densities of CD206^+^Iba1^+^ cells (221.00 cells/mm^2^ in LPS vs. 3.32 cells/mm^2^ in CTR, *p* < 0.0001) and CD16/32^+^Iba1^+^ cells (224.60 cells/mm^2^ in LPS vs. 0 cells/mm^2^ in CTR, *p* < 0.0001) were observed dispersed within the dorsal column at 3 dpi. The density of CD206^+^Iba1^+^ cells was further increased (270.20 cells/mm^2^ in LPS vs. 1.39 cells/mm^2^ in CTR, *p* < 0.0001) and concentrated within the demyelination area at 10 dpi, while CD16/32^+^Iba1^+^ cells was significantly reduced compared to the early stage (140.80 cells/mm^2^ in LPS vs. 0 cells/mm^2^ in CTR, *p* < 0.0001). Densities of both CD206^+^Iba1^+^ and CD16/32^+^Iba1^+^ cells were markedly downregulated to the control levels at 21 dpi (CD206^+^Iba1^+^: 24.33 cells/mm^2^ in LPS vs. 1.95 cells/mm^2^ of CTR, *p* > 0.05; CD16/32^+^Iba1^+^: 21.69 cells/mm^2^ in LPS vs. 0 cells/mm^2^ in CTR, *p* > 0.05). The density of CD206^+^Iba1^+^ cells in LPS mice remained higher than that in LPC mice until the decrease at the late stage (3 dpi: 221.00 cells/mm^2^ in LPS vs. 163.00 cells/mm^2^ in LPC, *p* < 0.05; 10dpi: 270.20 cells/mm^2^ in LPS vs. 244.70 cells/mm^2^ in LPC, *p* > 0.05; 21 dpi: 24.33 cells/mm^2^ in LPS vs. 96.10 cells/mm^2^ in LPC, *p* < 0.01). The density of CD16/32^+^Iba1^+^ cells was higher than that in LPC mice at the early stage but was reduced and lower than LPC during the middle and late stages (3 dpi: 224.60 cells/mm^2^ in LPS vs. 200.40 cells/mm^2^ in LPC, *p* < 0.05; 10 dpi: 140.80 cells/mm^2^ in LPS vs. 344.20 cells/mm^2^ in LPC, *p* < 0.0001; 21 dpi: 21.69 cells/mm^2^ in LPS vs. 45.43 cells/mm^2^ in LPC, *p* < 0.05). The proportion of M1 and M2 phenotypes in M/M were different comparing LPC and LPS mice along all time-points (Fig. 8e, f). There was no significant change in the proportions of CD206^+^Iba1^+^ and CD16/32^+^Iba1^+^ at the early and middle stages in the LPC-induced demyelination area (Fig. 8e). A substantial decrease was identified at 21 dpi in the proportion of CD16/32^+^Iba1^+^, resulting in a dramatic decrease in the M1/M2 ratio during the late stage. In LPS mice, the M1/M2 ratio was similar to that in LPC mice at 3 dpi but dramatically dropped at 10 dpi due to reduced CD16/32^+^Iba1^+^ cells and increased CD206^+^Iba1^+^ cells (Fig. 8f).

Similar to the patterns of CD206^+^Iba1^+^cells and CD16/32^+^Iba1^+^ cells, the densities of Arg1^+^Iba1^+^ and CD86^+^Iba1^+^ cells were significantly increased at 3 dpi after LPC injection (Arg1^+^Iba1: 214.50 cells/mm^2^ in LPC vs. 1.01 cells/mm^2^ in CTR, *p* < 0.0001; CD86^+^Iba1^+^: 140.90 cells/mm^2^ in LPC vs. 0.43 cells/mm^2^ in CTR, *p* < 0.0001), and both phenotypes were localized along the spared dorsal margins (Additional file 4: Figure S2 A-B). The Arg1^+^Iba1^+^ cells were restricted to the demyelination lesion core and surrounded by other Iba1^+^ cells like CD86^+^Iba1^+^ cells at 10 dpi, consistent with the localization of CD206^+^Iba1^+^ and CD16/32^+^Iba1^+^ cells at 10 dpi (Fig. 8a, b). Densities of both Arg1^+^Iba1^+^ and CD86^+^Iba1^+^ cells were greatly reduced at 21 dpi (Arg1^+^Iba1^+^: 26.71 cells/mm^2^ in LPC vs. 0.45 cells/mm^2^ in CTR, *p* **>** 0.05; CD86^+^Iba1^+^: 90.28 cells/mm^2^ in LPC vs. 1.73 cells/mm^2^ in CTR, *p* < 0.0001). In LPS mice, similar to those of CD206^+^Iba1^+^ and CD16/32^+^Iba1^+^ cells (Fig. 8a, b), Arg1^+^Iba1^+^ and CD86^+^Iba1^+^ cells were diffusely localized and increased from the early to middle stage and returned to the control levels during the late stage (Additional file 4: Figure S2 A-B). The density of Arg1^+^Iba1^+^ cells in LPS mice was higher than that in LPC mice until the robust decrease at the late stage (3 dpi: 415.20 cells/mm^2^ in LPS vs. 214.50 cells/mm^2^ in LPC, *p* < 0.0001; 10 dpi: 260.60 cells/mm^2^ in LPS vs. 164.50 cells/mm^2^ in LPC, *p* < 0.0001; 21 dpi: 19.49 cells/mm^2^ in LPS vs. 26.71 cells/mm^2^ in LPC, *p* **>** 0.05). The density of CD86^+^Iba1^+^ cells in LPS mice was lower than that in LPC mice at all stages (3 dpi: 34.65 cells/mm^2^ in LPS vs. 140.90 cells/mm^2^ in LPC, *p* < 0.0001; 10 dpi: 136.60 cells/mm^2^ in LPS vs. 266.90 cells/mm^2^ in LPC, *p* < 0.0001; 21 dpi: 21.25 cells/mm^2^ in LPS vs. 90.28 cells/mm^2^ in LPC, *p* < 0.0001) (Additional file 4: Figure S2 C and D). These changes in Arg1^+^Iba1^+^ (M2) and CD86^+^Iba1^+^ (M1) cells were consistent with those in CD206^+^Iba1^+^ (M2) and CD16/32^+^Iba1^+^ (M1) cells between LPC- and LPS-induced lesions.

The proportion of CD86^+^Iba1^+^ cells remained at similar levels until the late stage with a mild decrease (LPC: 0.44 at 10 dpi vs. 0.41 at 3 dpi, *p* > 0.05; 0.34 at 21 dpi vs. 0.44 at 10 dpi, *p* < 0.05). However, the proportion of Arg1^+^Iba1^+^ cells progressively declined from 10 to 21 dpi (LPC: 0.26 at 10 dpi vs. 0.54 at 3 dpi, *p* < 0.0001; 0.10 at 21 dpi vs. 0.26 at 10 dpi, *p* < 0.001) in the LPC mice (Additional file 4: Figure S2 E). In LPS mice, although the proportion of Arg1^+^Iba1^+^ cells progressively declined from 10 to 21 dpi, it still remained at relatively high levels during the middle stage (LPS: 0.66 at 10 dpi vs. 0.76 at 3 dpi, *p* < 0.05; 0.11 at 21 dpi vs. 0.66 at 10 dpi, *p* < 0.0001). In contrast, the proportion of CD86^+^Iba1^+^ cells rapidly increased to a relatively low level at 3 dpi, then dramatically increased at 10 dpi but returned to the early stage level at 21 dpi (LPS: 0.34 at 10 dpi vs. 0.07 at 3 dpi, *p* < 0.0001; 0.12 at 21 dpi vs. 0.34 at 10 dpi, *p* < 0.0001) in the LPS mice (Additional file 4: Figure S2 F).

### Correlations between M/M polarization, myelin integrity, and sensorimotor function

**Fig. 9:**
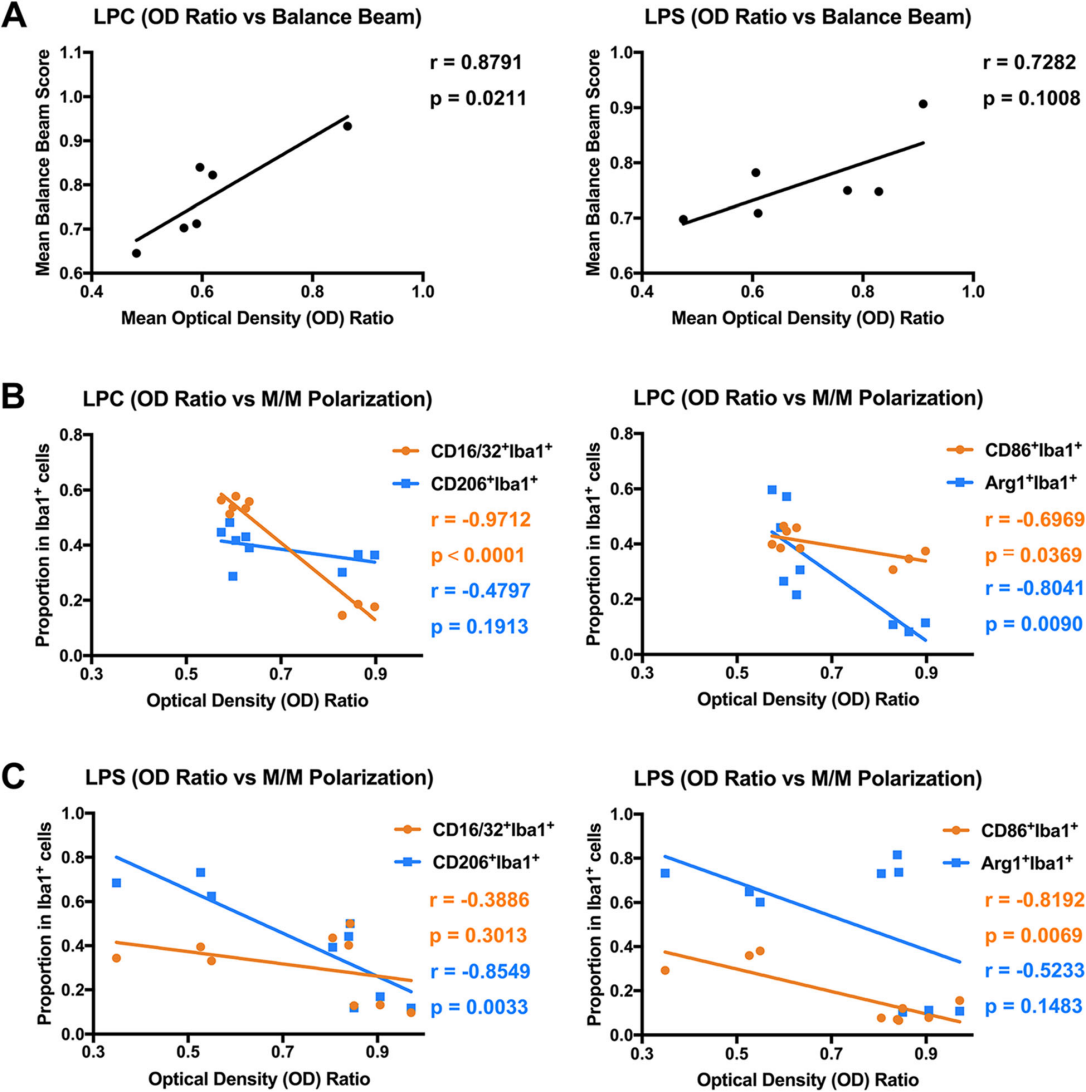
Correlations between demyelination severity, sensorimotor function, and M/M polarization. **a** Positive correlation between demyelination severity (OD ratio) and sensorimotor function (balance beam) was statistically significant in the LPC group (left; *r* = 0.8791, *p* = 0.0211), but not in the LPS group (right; *r* = 0.7282, *p* = 0.1008). **b** Negative correlations between demyelination (OD ratio) severity and M/M polarization in the LPC group (Left: CD16/32^+^Iba1^+^, *r* = − 0.9712, *p* < 0.0001; CD206^+^Iba1^+^, *r* = − 0.4797, *p* = 0.1913. Right: CD86^+^Iba1^+^, *r* = − 0.6969, *p* = 0.0369; Arg1Iba1^+^, *r* = − 0.8041, *p* = 0.0090). **c** Negative correlations between demyelination (OD ratio) severity and M/M polarization in the LPS group (Left: CD16/32^+^Iba1^+^, *r* = − 0.3886, *p* = 0.3013; CD206^+^Iba1^+^, *r* = − 0.8549, *p* = 0.0033. Right: CD86^+^Iba1^+^, *r* = − 0.8192, *p* = 0.0069; Arg1Iba1^+^, *r* = − 0.5233, *p* = 0.1483)

To identify the interdependence of demyelination severity and changes in sensorimotor function, correlations were measured between mean OD ratio and mean balance beam score after LPC or LPS injection (Fig. 9a). The size of a correlation coefficient (*r*) could be interpreted as very high positive or negative correlation (0.90 to 1.00 or − 0.90 to − 1.00), high positive or negative correlation (0.70 to 0.90 or − 0.70 to − 0.90), moderate positive or negative correlation (0.50 to 0.70 or − 0.50 to − 0.70), low positive or negative correlation (0.30 to 0.50 or − 0.30 to − 0.50), and little if any correlation (0.00 to 0.30 or 0.00 to − 0.30) [53, 54]. A high positive correlation was found in the LPC group (*r* = 0.8791, *p* = 0.0211), while a similar positive correlation trend was observed in the LPS group but with no statistical significance (*r* = 0.7282, *p* = 0.1008). Considering the distinct M/M polarization patterns between LPC- and LPS-induced demyelination, correlations between OD ratio and M1 (CD16/32^+^Iba1^+^ or CD86^+^Iba1^+^) and M2 (CD206^+^Iba1^+^ or Arg1^+^Iba1^+^) proportions were calculated in LPC and LPS groups, respectively (Fig. 9b, c). Significant negative correlation existed between OD ratio and CD16/32^+^Iba1^+^ (M1) or Arg1^+^Iba1^+^ (M2) proportion (very high in CD16/32^+^Iba1^+^: *r* = − 0.9712, *p* < 0.0001; high in Arg1^+^Iba1^+^: *r* = − 0.8041, *p* = 0.0090), while a moderate negative correlation existed between OD ratio and CD86^+^Iba1^+^ (M1) proportion (*r* = − 0.6969, *p* = 0.0369) in the LPC group. In contrast, high negative correlation was observed between OD ratio and CD86^+^Iba1^+^ (M1) or CD206^+^Iba1^+^ (M2) proportion (CD86^+^Iba1^+^: *r* = − 0.8192, *p* = 0.0069; CD206^+^Iba1^+^: r = − 0.8549, *p* = 0.0033) in the LPS group.

## Discussion

LPC- and LPS-induced focal demyelination mouse models in spinal white matter are widely used to investigate the cellular dynamic response during the demyelination-remyelination process. The LPC-induced demyelination lesion shows some features of the pattern I lesion (sharply demarcated lesion targeted on myelin; numerous oligodendrocytes reappear in central portion of the plaque) that occur in approximately 15% of biopsied MS patients [55], while the LPS model resembles the pattern III lesion (diffuse inflammatory demyelination) that is found in 26% of biopsied MS patients [26, 32, 33]. Although the temporal profile of these two focal MS models has been characterized [26, 29, 56], the dynamic response of glial cells during demyelination followed by spontaneous remyelination has not been clearly defined. In this study, we focused on the temporal and spatial features of M/M activation/polarization and OPC response in LPC- and LPS-induced focal demyelination lesions. We found that LPC and LPS induce special lesion patterns (Additional file 5: Figure S3). The unique lesion patterns and balance beam function are tightly correlated to specific M/M polarization probably due to different pathogenic mechanisms leading to demyelination.

LPC causes OL cell death and myelin loss followed by activation of secondary inflammation that may exaggerate the demyelination lesion. However, LPS initiates an inflammatory response that triggers the demyelination process. This difference in the pathological process between LPC and LPS is due to their distinct mechanisms leading to demyelination: LPC is a potent membrane-dissolving toxin while LPS activates inflammation via TLR-4 acting as an agonist [26, 29, 32]. Compared to the acute and intense lesion pattern elicited by LPC, the LPS-induced demyelination is progressive and relatively late (Fig. 2). Additional DTI imaging studies not only confirmed the temporal characteristics of the focal demyelination lesion by LFB staining but also proved a consistent and accurate in vivo imaging approach to monitor the progression of the demyelination-remyelination progress (Fig. 3). In accordance with the demyelination/histological patterns (OD ratio), balance beam performance showed similar temporal changes (Figs. 4 and 9a). This data indicated that fine sensorimotor function could be a sensitive assessment of demyelination in the dorsal columns of the spinal cord.

During the demyelination-remyelination process, a dynamic glial cell response occurs in the epicenter of the demyelination lesion, including activation of M/M and astrocytes, recruitment of OPCs, OPC proliferation, and OL differentiation. In LPC-induced focal lesions, adult OPCs aggregated along the adjacent GM and margin of the lesion and dramatically proliferated at the early stage, which migrated into the lesion epicenter and differentiated to initiate remyelination during the middle stage (Fig. 5 and Additional file 3: Figure S1 A). Meanwhile, LPC-mediated glial activation (astrocytes and M/M) as well as proliferation started in the early stage and reached its peak level during the middle stage, corresponding to secondary inflammation induced after the toxin-induced direct demyelination (Figs. 5 and Additional file 3: Figure S1 B and C). The M2 (CD206^+^ and Arg1^+^) was localized in the lesion epicenter with OPCs surrounded by M1 (CD16/32^+^ and CD86^+^) and activated astrocytes (Figs. 6 and 8 and Additional file 4: Figure S2). In contrast, OPCs, activated M/M (both M1 and M2), and astrocytes were distributed diffusely throughout the demyelination in the dorsal column WM and adjacent GM in LPS-induced lesions. OPC recruitment and glial activation dramatically increased at both early and middle stages but were significantly downregulated at the late stage (Figs. 5, 6, and 8, and Additional file 4: Figure S2).

Cytokines are a key link between the neuroinflammatory response and demyelination-remyelination process. The activated M/M and astrocytes are primary sources of the production of pro- and anti-inflammatory factors [12, 26, 57]. Significant upregulation of monocyte-secreted TNFα is detected in patients with stable MS [58]. Both in vitro and in vivo experimental studies showed that excessive TNFα causes myelin swelling, oligodendrocyte necrosis, and demyelination [59, 60]. Another pro-inflammatory cytokine IL-1β is also significantly released into the cerebrospinal fluid of MS patients in the active disease state [61], and higher levels are closely related to a more severe progression and poor prognosis of patients with a relapsing-remitting MS [62, 63]. Chronic expression of IL-1β in the rat striatum via adenovirus results in inflammatory toxicity and demyelination [62, 64]. As anti-inflammatory cytokines, TGF-β1 is reduced in serum but increased in the cerebrospinal fluid in MS patients [65] and IGF-1 is detected in activated astrocytes in active MS lesions [66] even though the level of IGF-1 in serum and cerebrospinal fluid are not changed in MS patients [67]. Therapeutic application of TGF-β1 or IGF-1 in animals showed a neuroprotective role in demyelination by suppressing pro-inflammatory cytokines, supporting OL differentiation, and promoting remyelination [68–71]. In our study, dramatic increases in pro-inflammatory cytokines were identified in the early stage of the LPS model and middle stage of the LPC model, in correlation with their different timing in the inflammatory response and M/M activation level (Fig. 7). The production of anti-inflammatory cytokines has gradually increased, which might be induced by the pro-inflammatory molecules, corresponding to the increased polarization into M2 and improved niche for the survival, recruitment, and differentiation of OPC/OL. The levels of pro-inflammatory cytokines like TNFα (from astrocytes and M/M (CD86^+^)) and IL-1β (only from M/M (CD86^+^)) [72] are increased in cerebrospinal fluid and serum of MS patients, with a positive correlation with disease severity [73, 74]. Despite the damaging effect of OL cell death and axonal degeneration [75, 76], pro-inflammatory cytokines may be beneficial for remyelination. TNFα and IL-1β promote the astrocytic synthesis of neurotrophic/growth factors like neurotrophins and ciliary neurotrophic factor, resulting in the improved survival and differentiation of OPC/OL [76–81]. TNFα is reported to improve OPC proliferation and remyelination via tumor necrosis factor receptor 2 (TNFR2) activation [73, 81], which might be related to an increased expression of chemokine C-X-C motif chemokine ligand 12 (CXCL12) from astrocytes [73]. IL-1β promotes remyelination by enhancing the mobilization of adult OPCs [82]. Moreover, IL-1β is found to induce the production of anti-inflammatory cytokine IGF-1, presumably from both activated M/M (CD206^+^ or Arg1^+^) and astrocytes, which promotes the accumulation and differentiation of OPCs and protects OL from cell death [83, 84]. Another important anti-inflammatory cytokine, TGF-β1, is released from activated M/M (CD206^+^ or Arg1^+^) and this signaling is accentuated by pro-inflammatory molecules to deactivate M/M activation and drive M/M polarization into M2 in combination with other anti-inflammatory cytokines (IL4 and IL10) [85–88]. It is noteworthy that the roles of these cytokines during the pathological process are interregulated, and the balance is dynamic and delicate. Therefore, it is crucial to boost the correct M/M phenotype at the right time with consideration of the dynamic balance of cytokines.

It has long been debated whether M/M activation is detrimental or beneficial in MS. M/M is a central player in surveillance and neuroinflammation [12, 57]. Focal accumulation of M/M is found at the sites of active demyelination and neurodegeneration [89–92]. Moreover, M/M in active MS lose homeostatic status and display M1 characteristics or an intermediate activation status, and restoration of M/M status is related to disease inactivity [89, 93]. The accumulation of M/M and the timing of M/M polarization affect the efficiency of remyelination, which might be related to the regulation of the OPC/OL response via the production of inflammation-relevant cytokines and growth/trophic factors [14, 16, 57, 77]. Our findings indicate that LPC- and LPS-induced demyelination models adopt different patterns of localization/timing of M/M activation and M/M polarization (Figs. 6, 8, and 9, and Additional file 4: Figure S2). In addition, different temporal OPC/OL responses during recruitment and differentiation may explain the different magnitudes of lesion severity and duration of the demyelination-remyelination process between LPC- and LPS-induced lesions (Fig. 5). A time lag of 7–10 days was observed in the demyelination LPS group and the M/M activation/M1-relevant pro-inflammatory cytokines (Fig. 7a, b) in the LPC group. These distinct time courses may be explained by the different timing of demyelination and inflammation between the LPC and LPS groups. Notably, the M1/M2-dominant phenotype was observed at the middle stage in the LPC lesions, while the M2/M1-dominant phenotype occurred at the early and middle stages in LPS lesions (Fig. 8 and Additional file 4: Figure S2). Both M1 and M2 play major roles in the LPC- and LPS-induced demyelination-remyelination process. However, specific profiles of M/M polarization are tightly correlated to the pathogenic mechanism leading to demyelination (Fig. 9b, c). A unique spatial change was identified in phenotype localization in the LPC group at 10 dpi (the timing for initial remyelination). The M2 phenotype was concentrated within the demyelination lesion core, surrounded by OPC/OL, astrocytes, and M1 phenotype. This regional arrangement suggests that, besides the polarization phenotype, the spatial change of M1 (outside; surveillance, barrier, pro-inflammation) and M2 (inside; regeneration, protection, anti-inflammation) could be another essential factor for OPC differentiation and initial remyelination. Although M1 is generally considered detrimental due to its roles in pro-inflammation, the findings in the phenotype spatial and temporal alternations, the relevant secreted cytokines, and the response of OPC/OL suggest that both M1 and M2 are critical to efficient remyelination. The balance and timing of M/M localization and polarization play essential roles in remodeling OPC behavior and the demyelination-remyelination process.

Of note, only female mice were used in the current study. MS is a sexually dimorphic disease with a female-to-male ratio as high as 3:1 [94, 95]. Sex-dependent differences in incidence, progression, and responses are reported in MS patients [96, 97] and experimental autoimmune encephalomyelitis rodent models [98, 99]. Female mice are more vulnerable to demyelination, which may be related to the shorter oligodendrocyte lifespan and higher myelin protein turnover [100]. Females are more easily to develop severe demyelination with stronger innate and adaptive immune responses, including greater M/M activation and phagocytic capacity, pro-inflammatory cytokine responses, and T cell proliferation and antibody responses [101]. The density of oligodendrocytes and myelin gene expression in male mice are greater than female mice [100, 102], which make them less vulnerable to demyelination insults. However, male mice have a higher expression of TLR-4 on M/M, which might mediate LPS to trigger severe inflammatory responses with more pro-inflammatory cytokine production [101, 103]. Detailed sexual dimorphism in glial and inflammatory responses are worthy of further investigation during the demyelination-remyelinaiton process.

## Conclusions

LPC and LPS induce distinctive temporal and spatial demyelination patterns: LPS produces diffuse demyelination lesions, and its peak of demyelination and functional decline occurs later than LPC. OPC/OL, astrocytes, and M/M are scattered throughout the LPS-induced demyelination lesions but are distributed in a layer-like pattern in the LPC-induced lesions. The specific M/M polarization is tightly correlated to specific pathology and sensorimotor function probably caused by different pathogenic mechanisms that leads to demyelination. The differences in M/M subpopulations and its localization are closely associated with the demyelination type and timing, reflecting the complex roles of M/M during the demyelination-remyelination process. The inflammatory response is a key feature of MS and a promising therapeutic target. These findings provide a fundamental knowledge on the pathological glial response in different types of demyelination lesions. These findings will benefit the strategic development in promoting/inhibiting certain inflammatory phenotypes at the appropriate time.

## Supplementary information

**Additional file 1:** **Table S1.** Antibodies for Immunohistochemistry.

**Additional file 2: Table S2.** TaqMan Gene Expression Assays (FAM Dye/MGB probe) for Real-time qRT-PCR.

**Additional file 3: Figure S1.** Temporal characteristics of glial cell proliferation following dorsal demyelination. (A-C) Representative photomicrographs of proliferative (A) OPCs (Pdgfrα^+^Ki67^+^), (B) M/M (Iba1^+^Ki67^+^), and (C) astrocytes (GFAP^+^Ki67^+^) in the dorsal demyelination area in the CTR, LPC, and LPS groups at 3, 10, and 21 dpi. Dashed white line indicates the margins of the dorsal columns. Representative double labelling is indicated by arrowhead and single labelling is indicated by arrow in the inset for better view. Scale bar, 50 μm for panel figure; 20 μm for inset. (D-F) Quantification of the cell density of proliferative (D) OPCs (Pdgfrα^+^Ki67^+^ cells/mm^2^), (E) M/M (Iba1^+^Ki67^+^ cells/mm^2^), and (F) astrocytes (GFAP^+^Ki67^+^ cells/mm^2^) within the dorsal column lesion site in the three groups at 3, 10, and 21 dpi. Cell densities was compared to the CTR group (** *p* < 0.01, *** *p* < 0.001, **** *p* < 0.0001) and the LPC group (× *p* < 0.05, × × × *p* < 0.001, × × × × *p* < 0.0001) at each given time-point; for each group, cell density was compared to 3 dpi (## *p* < 0.01, ### *p* < 0.001, #### *p* < 0.0001) and 10 dpi (+++ *p* < 0.001, ++++ *p* < 0.0001). (G, H) Proportions of proliferative OPCs (Pdgfrα^+^Ki67^+^), M/M (Iba1^+^Ki67^+^), and astrocytes (GFAP^+^Ki67^+^) in total proliferative cells (Ki67^+^) within the dorsal column at the lesion site in (G) LPC and (H) LPS groups at 3, 10, and 21 dpi. The cell proportion was compared to 3 dpi (# *p* < 0.05, ## *p* < 0.01, #### *p* < 0.0001) and 10 dpi (++ *p* < 0.01, ++++ *p* < 0.0001) for each group. Data were collected from three animals per group at each time-point (*n* = 3 mice).

**Additional file 4: Figure S2.** Temporal characteristics of M/M polarization and localization (Arg1^+^Iba1^+^ and CD86^+^Iba1^+^) following dorsal demyelination models. (A, B) Representative photomicrographs of (A) M2 (Arg1^+^Iba1^+^) and (B) M1 (CD86^+^Iba1^+^) at the dorsal demyelination area in the CTR, LPC, and LPS groups at 3, 10, and 21 dpi. Dashed white line indicates the margins of the dorsal columns. Representative double labelling is indicated by arrowhead and single labelling is indicated by arrow in the inset for better view. Scale bar, 50 μm for panel figure; 20 μm for inset. (C, D) Quantification of cell densities of (C) M2 (Arg1^+^Iba1^+^ cells/mm^2^) and (D) M1 (CD86^+^Iba1^+^ cells/mm^2^) within the dorsal columns at the lesion site in the three groups at 3, 10, and 21 dpi. Cell density was compared to the CTR group (* *p* < 0.05, **** *p* < 0.0001) and the LPC group (× × × × *p* < 0.0001) at each given time-point; for each group, cell density was compared to 3 dpi (## *p* < 0.01, #### *p* < 0.0001) and 10 dpi (++++ *p* < 0.0001). (E, F) Summary of the proportion of M2 (CD206^+^Iba1^+^, Arg1Iba1^+^) and M1 (CD16/32^+^Iba1^+^, CD86^+^Iba1^+^) in M/M (Iba1^+^) within the dorsal columns at the lesion site in (E) LPC and (F) LPS demyelination models at 3, 10, and 21 dpi. The cell proportion was compared to 3 dpi (# *p* < 0.05, #### *p* < 0.0001) and 10 dpi (+ *p* < 0.05, +++ *p* < 0.001, ++++ *p* < 0.0001) for each group. Data were collected from three animals per group at each time-point (n = 3 mice).

**Additional file 5: Figure S3.** Graphic summary of LPC- and LPS-induced distinctive temporal and spatial demyelination patterns. In contrast to LPC-induced robust demyelination with a lag in inflammation/glial response, LPS stimulated strong inflammation/glial response from the early stage and produced diffuse demyelination lesions with its peak demyelination and functional decline later than LPC. OPC/OL, astrocytes, and M/M were scattered throughout the LPS-induced demyelination lesion, but were distributed in a layer-like pattern in the LPC-induced lesion. OPCs populated and migrated into the lesion center to differentiate into mature OLs: OPC/OL were distributed dispersedly in the LPS-induced lesion; while in LPC-induced lesion, OPC/OL were localized in layers around M2. At the late stage, both LPC- and LPS- produced demyelination reached spontaneous remyelination with the restoration of inflammatory niche, a change in M/M subpopulations, and the recruitment/differentiation of OPC/OL into the lesion center. Specific M1/M2 polarization was closely associated with the demyelination-remyelination process, which might be related to the different mechanisms of LPC and LPS in causing inflammation and demyelination.

## Data Availability

The datasets generated and/or analyzed in the current study are available from the corresponding author on reasonable request.

## Abbreviations

AD: Axial diffusivity
ANOVA: Analysis of variance
Arg1: Arginase 1
CC1: Anti-adenomatous polyposis coli clone
CD: Cluster of differentiation
CNS: Central nervous system
CTR: Control
CXCL12: C-X-C motif chemokine ligand 12
dpi: Days post-injection
DTI: Diffusion tensor imaging
FA: Fractional anisotropy
GFAP: Glial fibrillary acidic protein
GST-π: Glutathione S-Transferase Pi
Iba1: Ionized calcium binding adaptor molecule 1
IGF-1: Insulin-like growth factor-1
IL-1β: Interleukin-1 beta
IOD: Integrated optical density
LFB: Luxol fast blue
LPC: L-α-Lysophosphatidylcholine
LPS: Lipopolysaccharide
M/M: Microglia/macrophages
MD: Mean diffusivity
MRI: Magnetic resonance imaging
MS: Multiple sclerosis
OD: Optical density
OL: Oligodendrocytes
Olig2: Oligodendrocyte Transcription Factor 2
OPC: Oligodendrocyte progenitor cell
PBS: Phosphate-buffered saline
Pdgfrα: Platelet-derived growth factor receptor alpha
qRT-PCR: Real-time quantitative reverse transcription polymerase chain reaction
RD: Radial diffusivity
RNA: Ribonucleic acid
ROI: Region of interest
SD: Standard deviation
TGF-β1: Transforming growth factor-beta 1
TLR: Toll-like receptor
TNFR2: Tumor necrosis factor receptor 2
TNFα: Tumor necrosis factor alpha
WM: White matter
