# Supplementary material for: Dynamic response of microglia/macrophage polarization following demyelination in mice
Source: J Neuroinflammation. 2019 Oct 17;16:188. doi: 10.1186/s12974-019-1586-1 (PMC6798513; doi:10.1186/s12974-019-1586-1)
Supplement: Supplementary file 1 — Additional file 1: Table S1. Antibodies for Immunohistochemistry. [file 12974_2019_1586_MOESM1_ESM.docx]

Table S1. Antibodies for Immunohistochemistry

| Antibody | Manufacturer | Dilution | Special treatment |
| --- | --- | --- | --- |
|  | | | |
|  | | | |
| Primary antibodies | | | |
| Oligodendrocyte-linage cells | | | |
|  |  |  |  |
| Olig2 (pAb) | Millipore/MAB9610 | 1:800 | Antigen retrieval |
| Pdgfra (pAb) | Novus Biologicals/AF1062 | 1:30 | Antigen retrieval |
| APC (CC-1; mAb) | Calbiochem/OP80 | 1:60 |  |
| GST-π (3/GST-π; mAb) | BD Biosciences/610718 | 1:500 |  |
|  | | | |
| Microglia/macrophage | | | |
|  |  |  |  |
| IbaI (pAb) | Wako/019-19741 | 1:500 |  |
| IbaI (pAb) | Novus Biologicals/NB100-1028 | 1:500 |  |
| CD68 (FA-11; mAb) | Biolegend/137002 | 1:50 |  |
| CD86 (GL1; mAb) | BD Biosciences/553689 | 1:100 |  |
| CD16/32 (2.4G2; mAb) | BD Biosciences/553141 | 1:100 |  |
| Arginase I (E-2; mAb) | Santa Cruz/sc-271430 | 1:100 | Antigen retrieval |
| CD206 (pAb) | R&D/AF2535 | 1:30 |  |
|  | | | |
| Astrocyte | | | |
|  |  |  |  |
| GFAP (GA5; mAb) | Millipore/MAB3402 | 1:1000 |  |
|  |  |  |  |
| Proliferation |  |  |  |
|  |  |  |  |
| Ki67 (SP6; mAb) | Thermo Scientific/RM-9106 | 1:200 | Antigen retrieval |
|  |  |  |  |
| Secondary antibodies |  |  |  |
|  |  |  |  |
| Goat anti-Rabbit IgG (H+L) Cross-Adsorbed Secondary Antibody, Alexa Fluor 594 | Invitrogen/A-11012 | 1:1000 |  |
| Donkey anti-Rabbit IgG (H+L) Highly Cross-Adsorbed Secondary Antibody, Alexa Fluor 594 | Invitrogen/A-21207 | 1:1000 |  |
|  |  |  |  |
| Donkey anti-Goat IgG (H+L) Cross-Adsorbed Secondary Antibody, Alexa Fluor 488 | Invitrogen/A-11055 | 1:1000 |  |
| Goat anti-Mouse IgG1 Cross-Adsorbed Secondary Antibody, Alexa Fluor 594 | Invitrogen/A-21125 | 1:1000 |  |
| Goat anti-Mouse IgG1 Cross-Adsorbed Secondary Antibody, Alexa Fluor 488 | Invitrogen/A-21121 | 1:1000 |  |
| Goat anti-Mouse IgG2b Cross-Adsorbed Secondary Antibody, Alexa Fluor 488 | Invitrogen/A-21141 | 1:1000 |  |
| Goat anti-Rat IgG (H+L) Cross-Adsorbed Secondary Antibody, Alexa Fluor 488 | Invitrogen/A-11006 | 1:1000 |  |
| Nucleic acid staining |  |  |  |
|  |  |  |  |
| DAPI | Sigma/D9542 | 1 μg/ml |  |
|  |  |  |  |

pAb: polyclonal antibody; mAb: monoclonal antibody; DAPI: 4',6-diamidino-2-phenylindole
