# Supplementary material for: Dynamic response of microglia/macrophage polarization following demyelination in mice
Source: J Neuroinflammation. 2019 Oct 17;16:188. doi: 10.1186/s12974-019-1586-1 (PMC6798513; doi:10.1186/s12974-019-1586-1)
Supplement: Supplementary file 2 — Additional file 2: Table S2. TaqMan Gene Expression Assays (FAM Dye/MGB probe) for Real-time qRT-PCR. [file 12974_2019_1586_MOESM2_ESM.docx]

Table S2. TaqMan Gene Expression Assays (FAM Dye/MGB probe) for Real-time qRT-PCR

| Gene Symbol | Target Gene | RefSeq | Assay ID |
| --- | --- | --- | --- |
|  | | | |
| Cytokines | | | |
|  |  |  |  |
| *TNFα* | Tumor necrosis factor | NM_001278601  NM_013693 | Mm00443258_m1 |
|  |  |  |  |
| *IL-1β* | Interleukin 1 beta | NM_008361 | Mm00434228_m1 |
| *TGF-β1* | Transforming growth factor, beta 1 | NM_011577 | Mm01178820_m1 |
|  |  |  |  |
| *IGF-1* | Insulin-like growth factor 1 | NM_001111274  NM_001111275  NM_001111276  NM_010512 | Mm00439560_m1 |
|  |  |  |  |
| Endogenous control | | | |
|  |  |  |  |
| *ACTB* | Actin, beta | NM_007393 | Mm00607939_s1 |
|  |  |  |  |
