# Supplementary figures and images for: Dynamic response of microglia/macrophage polarization following demyelination in mice
Source: J Neuroinflammation. 2019 Oct 17;16:188. doi: 10.1186/s12974-019-1586-1 (PMC6798513; doi:10.1186/s12974-019-1586-1)

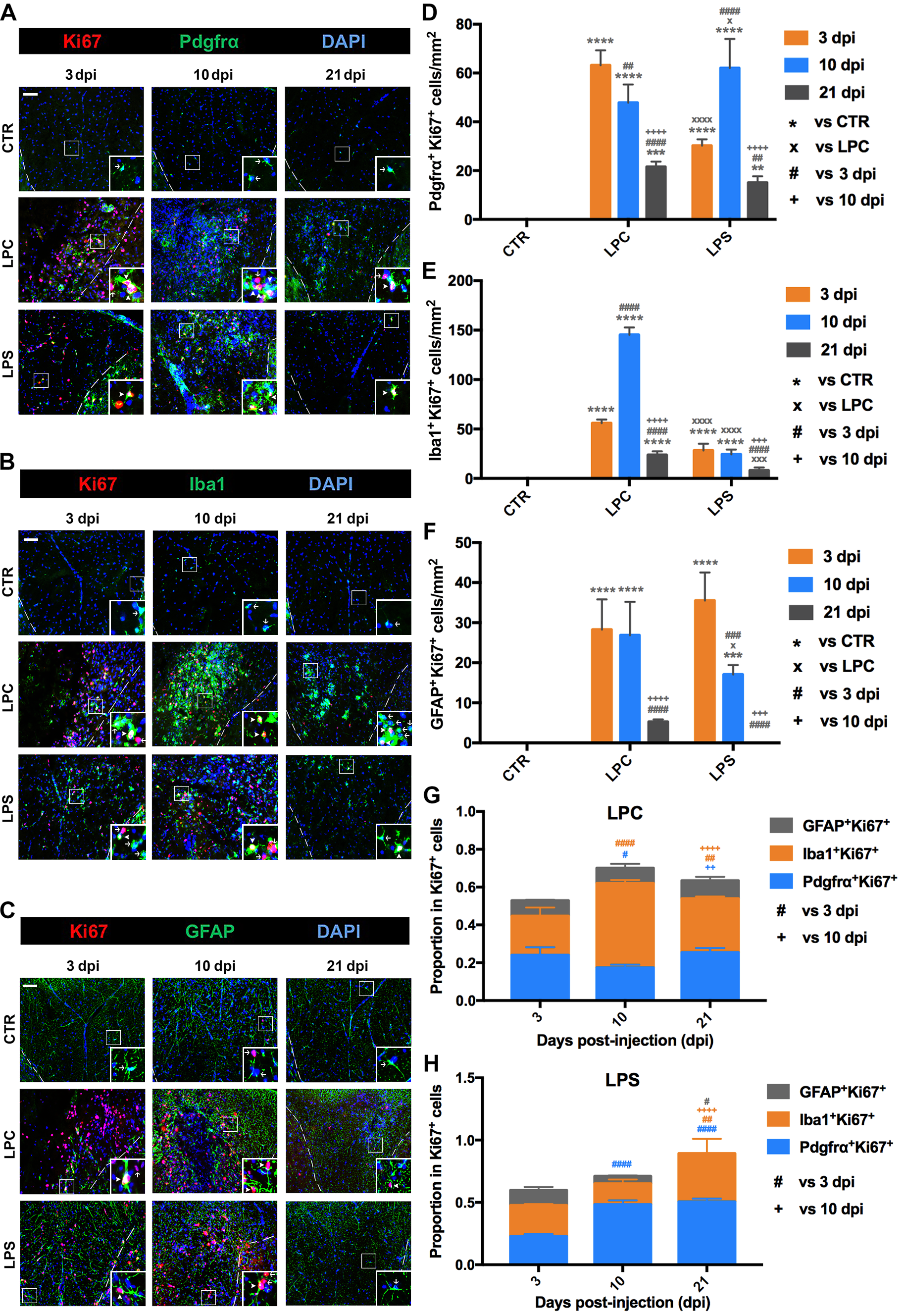

Supplement: Supplementary file 3 — Additional file 3: Figure S1. Temporal characteristics of glial cell proliferation following dorsal demyelination. (A-C) Representative photomicrographs of proliferative (A) OPCs (Pdgfrα+Ki67+), (B) M/M (Iba1+Ki67+), and (C) astrocytes (GFAP+Ki67+) in the dorsal demyelination area in the CTR, LPC, and LPS groups at 3, 10, and 21 dpi. Dashed white line indicates the margins of the dorsal columns. Representative double labelling is indicated by arrowhead and single labelling is indicated by arrow in the inset for better view. Scale bar, 50 μm for panel figure; 20 μm for inset. (D-F) Quantification of the cell density of proliferative (D) OPCs (Pdgfrα+Ki67+ cells/mm2), (E) M/M (Iba1+Ki67+ cells/mm2), and (F) astrocytes (GFAP+Ki67+ cells/mm2) within the dorsal column lesion site in the three groups at 3, 10, and 21 dpi. Cell densities was compared to the CTR group (** p < 0.01, *** p < 0.001, **** p < 0.0001) and the LPC group (× p < 0.05, × × × p < 0.001, × × × × p < 0.0001) at each given time-point; for each group, cell density was compared to 3 dpi (## p < 0.01, ### p < 0.001, #### p < 0.0001) and 10 dpi (+++ p < 0.001, ++++ p < 0.0001). (G, H) Proportions of proliferative OPCs (Pdgfrα+Ki67+), M/M (Iba1+Ki67+), and astrocytes (GFAP+Ki67+) in total proliferative cells (Ki67+) within the dorsal column at the lesion site in (G) LPC and (H) LPS groups at 3, 10, and 21 dpi. The cell proportion was compared to 3 dpi (# p < 0.05, ## p < 0.01, #### p < 0.0001) and 10 dpi (++ p < 0.01, ++++ p < 0.0001) for each group. Data were collected from three animals per group at each time-point (n = 3 mice). [file 12974_2019_1586_MOESM3_ESM.tif]

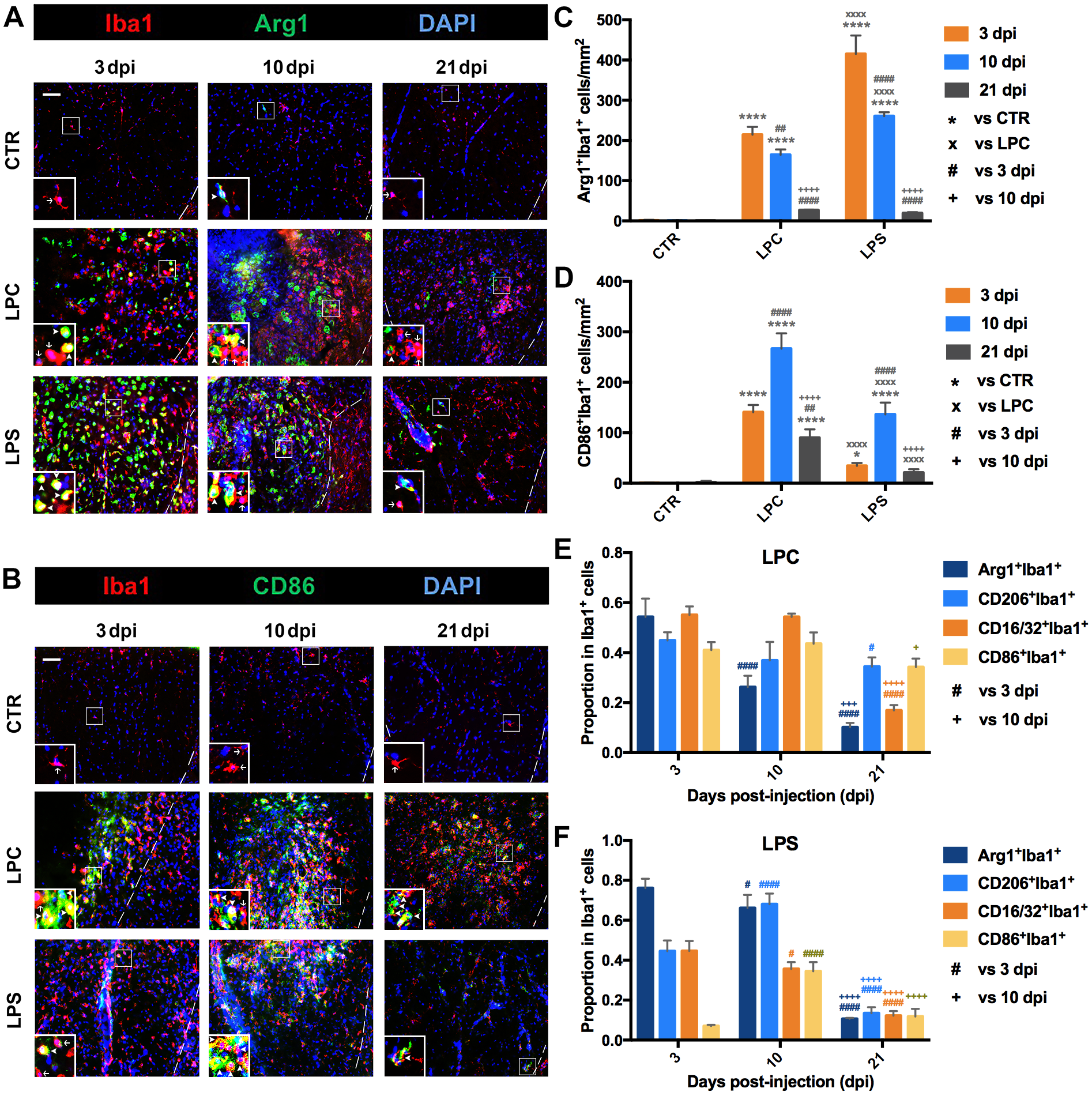

Supplement: Supplementary file 4 — Additional file 4: Figure S2. Temporal characteristics of M/M polarization and localization (Arg1+Iba1+ and CD86+Iba1+) following dorsal demyelination models. (A, B) Representative photomicrographs of (A) M2 (Arg1+Iba1+) and (B) M1 (CD86+Iba1+) at the dorsal demyelination area in the CTR, LPC, and LPS groups at 3, 10, and 21 dpi. Dashed white line indicates the margins of the dorsal columns. Representative double labelling is indicated by arrowhead and single labelling is indicated by arrow in the inset for better view. Scale bar, 50 μm for panel figure; 20 μm for inset. (C, D) Quantification of cell densities of (C) M2 (Arg1+Iba1+ cells/mm2) and (D) M1 (CD86+Iba1+ cells/mm2) within the dorsal columns at the lesion site in the three groups at 3, 10, and 21 dpi. Cell density was compared to the CTR group (* p < 0.05, **** p < 0.0001) and the LPC group (× × × × p < 0.0001) at each given time-point; for each group, cell density was compared to 3 dpi (## p < 0.01, #### p < 0.0001) and 10 dpi (++++ p < 0.0001). (E, F) Summary of the proportion of M2 (CD206+Iba1+, Arg1Iba1+) and M1 (CD16/32+Iba1+, CD86+Iba1+) in M/M (Iba1+) within the dorsal columns at the lesion site in (E) LPC and (F) LPS demyelination models at 3, 10, and 21 dpi. The cell proportion was compared to 3 dpi (# p < 0.05, #### p < 0.0001) and 10 dpi (+ p < 0.05, +++ p < 0.001, ++++ p < 0.0001) for each group. Data were collected from three animals per group at each time-point (n = 3 mice). [file 12974_2019_1586_MOESM4_ESM.tif]

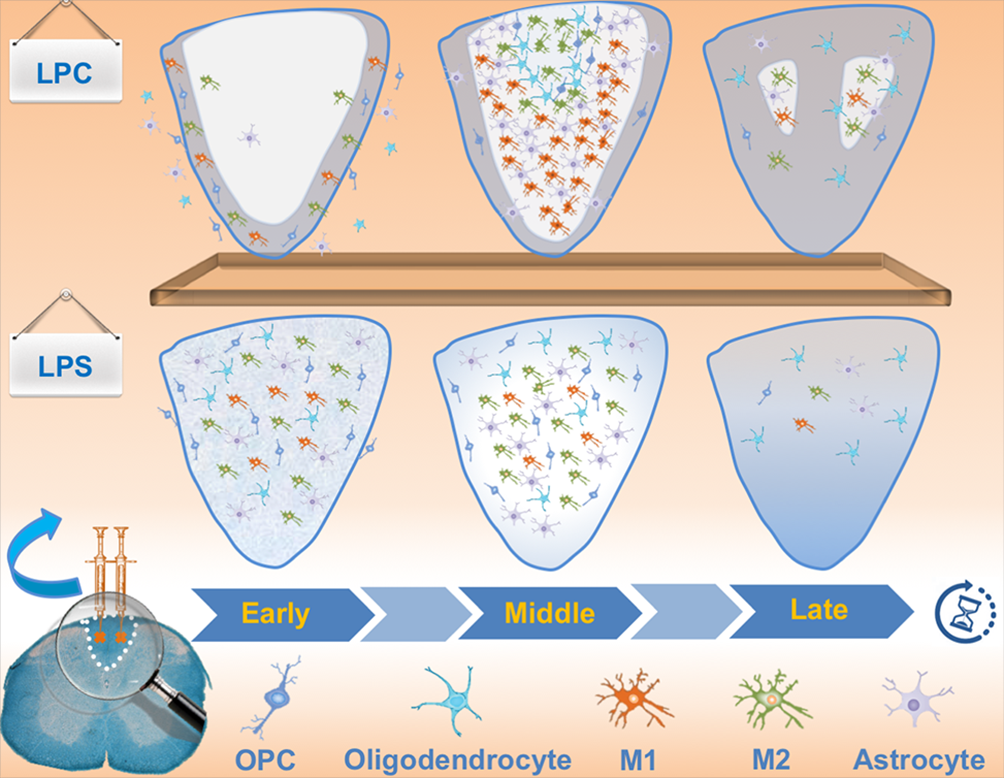

Supplement: Supplementary file 5 — Additional file 5: Figure S3. Graphic summary of LPC- and LPS-induced distinctive temporal and spatial demyelination patterns. In contrast to LPC-induced robust demyelination with a lag in inflammation/glial response, LPS stimulated strong inflammation/glial response from the early stage and produced diffuse demyelination lesions with its peak demyelination and functional decline later than LPC. OPC/OL, astrocytes, and M/M were scattered throughout the LPS-induced demyelination lesion, but were distributed in a layer-like pattern in the LPC-induced lesion. OPCs populated and migrated into the lesion center to differentiate into mature OLs: OPC/OL were distributed dispersedly in the LPS-induced lesion; while in LPC-induced lesion, OPC/OL were localized in layers around M2. At the late stage, both LPC- and LPS- produced demyelination reached spontaneous remyelination with the restoration of inflammatory niche, a change in M/M subpopulations, and the recruitment/differentiation of OPC/OL into the lesion center. Specific M1/M2 polarization was closely associated with the demyelination-remyelination process, which might be related to the different mechanisms of LPC and LPS in causing inflammation and demyelination. [file 12974_2019_1586_MOESM5_ESM.tiff]
